# Intake of *n*-3 polyunsaturated fatty acids in childhood, *FADS* genotype and incident asthma

**DOI:** 10.1183/13993003.03633-2020

**Published:** 2021-09-02

**Authors:** Mohammad Talaei, Emmanouela Sdona, Philip C. Calder, Louise R. Jones, Pauline M. Emmett, Raquel Granell, Anna Bergström, Erik Melén, Seif O. Shaheen

**Affiliations:** 1 Institute of Population Health Sciences, Barts and The London School of Medicine and Dentistry, Queen Mary University of London, London, UK; 2 Institute of Environmental Medicine, Karolinska Institute, Stockholm, Sweden; 3 Human Development and Health, Faculty of Medicine, University of Southampton, Southampton, UK; 4 NIHR Southampton Biomedical Research Centre, University Hospital Southampton NHS Foundation Trust and University of Southampton, Southampton, UK; 5 Centre for Academic Child Health, Population Health Sciences, Bristol Medical School, University of Bristol, Bristol, UK; 6 MRC Integrative Epidemiology Unit, Population Health Sciences, Bristol Medical School, University of Bristol, Bristol, UK; 7 Centre for Occupational and Environmental Medicine, Region Stockholm, Stockholm, Sweden; 8 Dept of Clinical Science and Education, Södersjukhuset, Karolinska Institute, Stockholm, Sweden; 9 Sachs’ Children and Youth Hospital, Södersjukhuset, Stockholm, Sweden

## Abstract

Longitudinal evidence on the relation between dietary intake of *n*-3 (ω-3) very-long-chain polyunsaturated fatty acids, *i.e.* eicosapentaenoic acid (EPA) and docosahexaenoic acid (DHA), in mid-childhood and asthma risk is scarce. We aimed to investigate whether a higher intake of EPA and DHA from fish in childhood is associated with a lower risk of incident asthma.

In the Avon Longitudinal Study of Parents and Children, dietary intakes of EPA and DHA from fish were estimated by food frequency questionnaire at 7 years of age. We used logistic regression, controlling for confounders, to analyse associations between intake of EPA and DHA (quartiles) and incidence of doctor-diagnosed asthma at age 11 or 14 years, and explored potential effect modification by a fatty acid desaturase (*FADS*) polymorphism (rs1535). Replication was sought in the Swedish BAMSE birth cohort.

There was no evidence of association between intake of EPA plus DHA from fish and incident asthma overall (n=4543). However, when stratified by *FADS* genotype, the odds ratio comparing the top *versus* bottom quartile among the 2025 minor G allele carriers was 0.49 (95% CI 0.31–0.79; p_trend_=0.006), but no inverse association was observed in the homozygous major A allele group (OR 1.43, 95% CI 0.83–2.46; p_trend_=0.19) (p_interaction_=0.006). This gene–nutrient interaction on incident asthma was replicated in BAMSE.

In children with a common *FADS* variant, higher intake of EPA and DHA from fish in childhood was strongly associated with a lower risk of incident asthma up to mid-adolescence.

## Abstract 2

**In children with a common fatty acid desaturase (*FADS*) variant, higher intake of eicosapentaenoic acid (EPA) and docosahexaenoic acid (DHA) from fish in mid-childhood was strongly associated with a lower risk of incident asthma up to mid-adolescence** https://bit.ly/2M4VxO6

## Introduction

A substantial body of epidemiological evidence has implicated diet early in the life course in the aetiology of asthma and other allergic diseases. However, most evidence in children comes from cross-sectional studies, thus limiting causal inference [1–3]. Fish intake has attracted particular interest, as fish is a rich source of the *n*-3 (ω-3) very-long-chain polyunsaturated fatty acids (VLC-PUFAs), *i.e.* eicosapentaenoic acid (EPA) and docosahexaenoic acid (DHA), which have anti-inflammatory effects [4–6]. Other nutrients in fish, such as vitamin D and selenium, may also protect against asthma risk [7].

The few longitudinal studies investigating associations between fish and *n*-3 VLC-PUFA intake and asthma and allergic diseases have focused on exposures during pregnancy or infancy [8]; one birth cohort reported no association between fish intake in mid-childhood and subsequent asthma, but did not investigate associations with *n*-3 VLC-PUFA intake [9]. In the Avon Longitudinal Study of Parents and Children (ALSPAC), 25% of children either developed new asthma or had asthma that remitted or persisted between 7 and 14 years of age [10]; dietary intake of fish and *n*-3 VLC-PUFAs in childhood could play an important role in influencing asthma risk at this stage of life. Moreover, any beneficial effects of these exposures on asthma might be most apparent in certain subgroups. Endogenous production of VLC-PUFAs depends on the efficiency of conversion of precursor fatty acids by fatty acid desaturase (FADS) [5]. The minor G allele of a *FADS* single nucleotide polymorphism (SNP), rs1535, predicts lower plasma EPA and DHA concentrations in a meta-analysis of genome-wide association studies [11] and in mothers taking part in a randomised controlled trial (RCT) of fish oil supplementation in pregnancy [12]. In the latter RCT, a beneficial effect of supplementation on the offspring's risk of asthma was greatest in children of mothers who carried the G allele [12], suggesting that exogenous supply of pre-formed EPA and DHA (*e.g.* from fish or from fish oil supplements) is necessary to achieve both a high status and the health benefits of EPA and DHA in individuals with the G allele of this *FADS* SNP.

In this study, we investigated the associations of intake of fish and *n*-3 VLC-PUFAs from fish at 7 years of age with incident asthma up to mid-adolescence. We also explored whether these associations were modified by child's *FADS* genotype, in order to strengthen causal inference.

## Methods

### Study population

ALSPAC is a population-based birth cohort that recruited predominantly White pregnant women resident in the former county of Avon in the UK with expected dates of delivery from 1 April 1991 to 31 December 1992 (14 541 pregnancies). The cohort has been followed since birth with annual questionnaires and, since age 7 years, with objective measures in annual research clinics. The study protocol has been described previously [13, 14] and further information can be found at www.alspac.bris.ac.uk, which contains details of all the data that are available (www.bristol.ac.uk/alspac/researchers/our-data). Ethics approval was obtained from the ALSPAC Ethics and Law Committee (Institutional Review Board approval 00003312) and the Local National Health Service Research Ethics Committees. Informed consent for the use of data collected *via* questionnaires and clinics was obtained from participants following the recommendations of the ALSPAC Ethics and Law Committee at the time.

### Exposure assessment

We used dietary information collected by food frequency questionnaire (FFQ) at 54 months (∼4 years) and 81 months (∼7 years) of age, which was completed by the child's mother or the main carer. The FFQ included questions about usual consumption of up to 56 food groups and 12 drinks, with five frequency options ranging from “never or rarely” to “more than once a day” [15]. Fish intake was covered by five items: shellfish, white fish in breadcrumbs or batter, white fish without coating, tuna and oily fish (details in the supplementary material). Total energy and nutrient intakes were calculated by multiplying the estimated food intake (g·day^−1^) by the estimated nutrient content from UK food composition tables [16, 17] and summing this across all the foods consumed. Fatty acid composition of fish was based on profiles of typical British species [16]. Accordingly, daily intakes of EPA and DHA from fish, total *n*-6 fatty acids and arachidonic acid (an *n*-6 PUFA which also depends on FADS for endogenous production) were estimated. Maternal intake of EPA and DHA at 32 weeks of gestational age was also estimated similarly by FFQ.

### Outcome assessment

Our primary outcome of interest was incident asthma. At 91 months (∼7.5 years), 128 months (∼11 years) and 166 months (∼14 years) of age, we defined current doctor-diagnosed asthma based on whether mothers responded positively to the question “Has a doctor ever actually said that your study child has asthma?”, and to at least one of the concurrent following questions that asked if the child had had wheezing, wheezing and whistling in the chest, asthma or asthma medication in the last 12 months. Among those children who were not identified as having current doctor-diagnosed asthma at 7.5 years, we defined those with current doctor-diagnosed asthma at 11 or 14 years as cases of incident asthma. Further details about secondary outcomes are available in the supplementary material.

### Genotyping

Among 20 SNPs related to *n*-3 metabolism in the literature, we selected a SNP in the *FADS2* gene, rs1535, as our main candidate variant because of prior strong evidence that it predicts blood levels of EPA and DHA [11] and also modifies the effect of fish oil supplementation [12]. It was imputed with 0.999 imputation quality using the 1000 Genomes reference panel (Phase 1, Version 3) (further details are available in the supplementary material). Participants with genetic evidence of non-European ancestry were excluded before imputation.

### Statistical analysis

**FIGURE 1:**
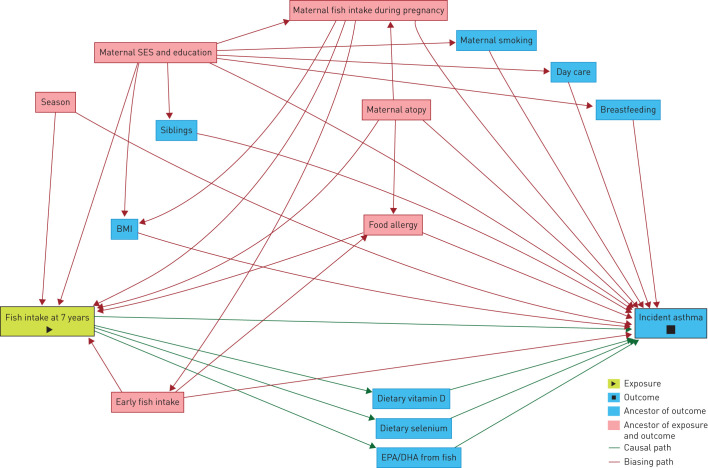
Directed acyclic graph to study covariates and potential structural confounding bias for the association between child's fish intake at 7 years and incident asthma risk. SES: socioeconomic status; BMI: body mass index; EPA: eicosapentaenoic acid; DHA: docosahexaenoic acid.

Among 8140 children with dietary data available at 7 years, data were complete on incident asthma for 4543 (supplementary figure E1). We used logistic regression to examine associations of intakes of fish, and EPA and DHA from fish (in quartiles), with incident asthma using the lowest quartile of intake as the reference category. Linear trend was tested by including median intake of quartiles as a pseudo-continuous variable in the models. We selected known potential confounding factors from the existing literature [18] and then refined our selection by using a directed acyclic graph approach (figure 1) [19]. Details of multivariable models and covariates are explained in the supplementary material.

We carried out stratified analyses *a priori* to explore potential modification of dietary associations by *FADS* genotype (rs1535: major A allele homozygous (AA) *versus* heterozygotes plus homozygous for minor G allele (GA/GG); the latter two genotypes were combined for analysis because the number of GG individuals was small: n=393 (10.8% of total) with only 29 cases of incident asthma). Distribution of allele frequencies for rs1535 was tested for deviation from the Hardy–Weinberg equilibrium using a likelihood ratio test (p=0.26). Potential interactions were assessed by testing the cross-product term of *FADS* genotype with quartiles (median values) as a continuous variable in the regression model.

As *FADS* genes are also involved in the *n*-6 pathway, leading to the production of arachidonic acid with pro-inflammatory effects, we also explored interactions between intake of total *n*-6 and arachidonic acid and rs1535 on asthma. We also assessed the relationship of cumulative exposure to EPA plus DHA, defined as being consistently in the top or bottom quartiles of intake at 4 and 7 years of age, with incident asthma. Dietary information at 4 years was not considered as a primary exposure because a lack of asthma diagnosis at this young age meant that subsequent incident asthma could not be defined. We also explored the interaction between maternal intake of EPA plus DHA during pregnancy and maternal *FADS* genotype on incident asthma at 11 or 14 years. Finally, we carried out several sensitivity analyses including further adjustments, exclusions, restricted cubic spline analysis to examine the dose–response relationship and inverse probability weighting to correct for potential loss-to-follow-up bias [20] (further details can be found in the supplementary material). All statistical analyses were carried out using Stata version 14.2 (StataCorp, College Station, TX, USA).

### Replication cohort

We used the Swedish population-based birth cohort study BAMSE (Swedish abbreviation for Children, Allergy, Milieu, Stockholm, Epidemiology) to replicate the main ALSPAC findings. 4089 infants, born during 1994–1996 in Stockholm, were enrolled in the BAMSE cohort and have been followed repeatedly [21, 22]. At the 8-year clinical examination parents were asked to fill in a FFQ containing questions about 98 foods and beverages frequently consumed in Sweden, including six questions on fish intake [23]. Intakes of EPA and DHA from fish were estimated using composition values obtained from the Swedish National Food Administration Database [24]. At age 8, 12 and 16 years, we defined current doctor-diagnosed asthma, very similarly to ALSPAC, and accordingly cases of incident asthma were determined at 12 or 16 years (n=2138). We used a similar analytic approach to that used in ALSPAC (further details in the supplementary material).

## Results

Median (interquartile range) intake of fish in ALSPAC was estimated as 24.3 (11.1–38.6) g·day^−1^ and mainly comprised white fish (on average 74.6% of total fish intake) followed by tuna (18.4%). Intakes of EPA and DHA from fish were 11.2 (5.99–24.0) and 17.6 (9.77–41.4) mg·day ^−1^, respectively, and were very highly correlated (r>0.95), so we focused our main analyses on combined intakes of EPA plus DHA. Table 1 shows characteristics of children and their mothers across quartiles of child's EPA plus DHA intake. Children with higher intakes of EPA plus DHA from fish at 7 years of age were more likely to be female, and had higher total energy intake, a generally more health-conscious dietary pattern and higher supplement use. These children were also more likely to have been exclusively breastfed by the third month of life, to have consumed fish before 6 months and to have a history of food allergy by 7 years of age. Mothers of children who had higher intakes of EPA plus DHA from fish were older, more educated, less likely to live in council rented houses and had a higher intake of EPA plus DHA from fish during late pregnancy. Among children with data on fish intake, 3370 (56.3%) carried the minor allele of the *FADS* genotype (rs1535). There was no evidence of a difference in background characteristics between AA and GA/GG genotype groups except for a higher tendency to the health-conscious dietary pattern in the AA group (supplementary table E1).

**TABLE 1:** Participant characteristics according to quartiles of eicosapentaenoic acid (EPA) plus docosahexaenoic acid (DHA) intake from fish at 7 years^#^ of age in the Avon Longitudinal Study of Parents and Children

	**Quartiles of EPA plus DHA intake from fish**				**p-value**
	**Quartile 1**	**Quartile 2**	**Quartile 3**	**Quartile 4**	
**Participants**	1050 (23.1)	1028 (22.6)	1325 (29.2)	1140 (25.1)	
**EPA plus DHA intake mg·day^−1^**	5.46±4.49	21.6±2.46	42.4±11.4	129±77.8	
**Male**	540 (51.4)	543 (52.8)	590 (44.5)	560 (49.1)	<0.001
**Total energy intake kJ·day^−1^**	7084±1653	7486±1649	7696±1660	7952±1772	<0.001
**BMI kg·m^−2^**	16.1±1.9	16.2±1.9	16.1±1.9	16.1±2.0	0.74
**BMI at 13.5 years kg·m^−2^**	20.2±3.30	20.4±3.42	20.1±3.20	20.0±3.12	0.11
**Health-conscious dietary pattern score**	−0.14±1.08	−0.28±0.80	−0.01±0.84	0.43±0.97	<0.001
**Any supplement use**	360 (34.3)	329 (32.0)	425 (32.1)	431 (37.8)	0.01
**Season of dietary information collection**					0.52
Winter	286 (27.2)	283 (27.5)	326 (24.6)	285 (25.0)	
Spring	323 (30.8)	306 (29.8)	389 (29.4)	338 (29.6)	
Summer	271 (25.8)	275 (26.8)	381 (28.8)	340 (29.8)	
Autumn	160 (15.2)	158 (15.4)	212 (16.0)	166 (14.6)	
Missing	10 (1.0)	6 (0.6)	17 (1.3)	11 (1.0)	
**Breastfeeding at 3 months**					<0.001
Never	177 (16.9)	196 (19.1)	189 (14.3)	114 (10.0)	
Stopped/non-exclusive	466 (44.4)	486 (47.3)	630 (47.5)	522 (45.8)	
Exclusive	359 (34.2)	313 (30.4)	451 (34.0)	453 (39.7)	
Missing	48 (4.6)	33 (3.2)	55 (4.2)	51 (4.5)	
**Age at fish introduction**					<0.001
≥9 months	316 (30.1)	207 (20.1)	264 (19.9)	168 (14.7)	
6– <9 months	276 (26.3)	288 (28.0)	372 (28.1)	280 (24.6)	
<6 months	450 (42.9)	529 (51.5)	687 (51.8)	686 (60.2)	
Missing	8 (0.8)			6 (0.5)	
**History of food allergy**	177 (16.9)	148 (14.4)	196 (14.8)	224 (19.6)	0.002
**Childcare by day nursery at 15 months**					0.048
No	950 (90.5)	937 (91.1)	1172 (88.5)	1018 (89.3)	
Yes	65 (6.2)	60 (5.8)	119 (9.0)	92 (8.1)	
Missing	35 (3.3)	31 (3.0)	34 (2.6)	30 (2.6)	
**Older siblings**	585 (55.7)	527 (51.3)	658 (49.7)	583 (51.1)	0.03
**Younger siblings**	487 (46.4)	533 (51.8)	735 (55.5)	581 (51.0)	<0.001
***FADS* genotype (rs1535)**					0.38
AA	356 (42.9)	351 (43.1)	472 (43.8)	429 (47.1)	
GA	385 (46.4)	383 (47.0)	482 (44.7)	382 (41.9)	
GG	88 (10.6)	81 (9.9)	124 (11.5)	100 (11.0)	
**Maternal factors**					
Age years	29.5±4.5	29.3±4.5	29.2±4.3	29.9±4.3	<0.001
Education					<0.001
Secondary or vocational	217 (20.7)	217 (21.1)	233 (17.6)	156 (13.7)	
O-level	342 (32.6)	392 (38.1)	490 (37.0)	337 (29.6)	
A-level or degree	482 (45.9)	404 (39.3)	591 (44.6)	629 (55.2)	
Missing	9 (0.9)	15 (1.5)	11 (0.8)	18 (1.6)	
Housing tenure during pregnancy					0.049
Mortgaged/owned	879 (83.7)	863 (83.9)	1138 (85.9)	979 (85.9)	
Council rented	61 (5.8)	73 (7.1)	57 (4.3)	48 (4.2)	
Non-council rented	67 (6.4)	50 (4.9)	68 (5.1)	70 (6.1)	
Missing	43 (4.1)	42 (4.1)	62 (4.7)	43 (3.8)	
Financial difficulty					0.07
No	891 (84.9)	898 (87.4)	1113 (84.0)	971 (85.2)	
Yes	153 (14.6)	125 (12.2)	211 (15.9)	163 (14.3)	
Missing	6 (0.6)	5 (0.5)		6 (0.5)	
Ethnicity					0.004
White	1028 (97.9)	1000 (97.3)	1302 (98.3)	1093 (95.9)	
Non-White	13 (1.2)	10 (1.0)	10 (0.8)	26 (2.3)	
Missing	9 (0.9)	18 (1.8)	13 (1.0)	21 (1.8)	
History of atopy					0.45
No	529 (50.4)	551 (53.6)	718 (54.2)	595 (52.2)	
Yes	485 (46.2)	444 (43.2)	563 (42.5)	497 (43.6)	
Missing	36 (3.4)	33 (3.2)	44 (3.3)	48 (4.2)	
Smoking when child 7 years old					0.50
No	864 (82.3)	821 (79.9)	1085 (81.9)	929 (81.5)	
Yes	162 (15.4)	168 (16.3)	205 (15.5)	179 (15.7)	
Missing	24 (2.3)	39 (3.8)	35 (2.6)	32 (2.8)	
EPA plus DHA intake from fish at 32 weeks of gestation mg·day^−1^	91.4±101	107±105	136±116	174±132	<0.001

Data are presented as n (%) or mean±sd, unless otherwise stated. BMI: body mass index; FADS: fatty acid desaturase. ^#^: child characteristics pertain to 7 years of age unless otherwise stated.

### Fish intake

We did not find any evidence of association between fish intake at 7 years and incident asthma at 11 or 14 years (n=393) in the whole study sample (n=4543). However, when stratified by *FADS* genotype, a higher intake of fish was associated with a lower risk of incident asthma in the GA/GG group, but not in the AA group (table 2). The inverse association in the minor allele group was substantially attenuated when we further adjusted for intake of EPA plus DHA (adjusted OR comparing top *versus* bottom quartile 0.86, 95% CI 0.48–1.54; p_trend_=0.57), but not when adjusted for intake of vitamin D (OR 0.66, 95% CI 0.41–1.05; p_trend_=0.08) or selenium (OR 0.61, 95% CI 0.37–0.99; p_trend_=0.05).

**TABLE 2:** Odds ratio for incident asthma at 11 or 14 years according to intake of fish at 7 years of age, stratified by child's fatty acid desaturase (*FADS*) genotype in the Avon Longitudinal Study of Parents and Children

	**Quartiles of fish intake**				**p_trend_- value^#^**	**p_interaction_- value**
	**Quartile 1**	**Quartile 2**	**Quartile 3**	**Quartile 4**		
**Median (IQR) intake g·day^−1^**	6.07 (0.00–8.57)	14.6 (13.7–20.4)	27.2 (24.3–29.3)	46.5 (40.4–58.6)		
**Cases/non-cases**	104/1034	55/586	138/1518	98/1086		
Model 1	1.00	0.92 (0.65–1.30)	0.86 (0.66–1.13)	0.83 (0.61–1.11)	0.21	
Model 2	1.00	0.92 (0.65–1.29)	0.86 (0.65–1.12)	0.82 (0.61–1.11)	0.20	
Model 3	1.00	0.94 (0.67–1.33)	0.87 (0.66–1.14)	0.83 (0.62–1.13)	0.22	
***FADS* genotype (rs1535): AA**						
Cases/non-cases	28/360	24/198	61/540	32/385		
Model 3	1.00	1.67 (0.92–3.02)	1.41 (0.87–2.30)	1.06 (0.61–1.85)	0.81	
***FADS* genotype (rs1535): GA/GG**						
Cases/non-cases	54/456	23/271	55/671	39/491		
Model 3	1.00	0.66 (0.39–1.12)	0.64 (0.43–0.97)	0.59 (0.37–0.93)	0.03	0.22

Data are presented as n/n or OR (95% CI), unless otherwise stated. IQR: interquartile range. **^#^**: linear trend was tested by treating the median values of quartiles as a continuous variable. Multivariable model 1: sex and total energy intake at 7 years. Multivariable model 2: further adjusted for maternal education, housing tenure during pregnancy, financial difficulty during pregnancy and maternal ethnicity. Multivariable model 3: further adjusted for maternal history of atopic disease, maternal age at delivery, exclusive breastfeeding, childcare by day nursery at 15 months of age, maternal smoking, older sibling, younger sibling and season when the food frequency questionnaire was completed.

### EPA and DHA intake from fish

Intakes of EPA plus DHA from fish were not significantly associated with risk of incident asthma overall. However, when we stratified by *FADS* genotype, strong inverse associations were observed in the GA/GG group, with evidence of a dose–response relationship, but not in the AA group (p_interaction_=0.006) (table 3 and supplementary figure E2). In the GA/GG group, the proportion of children developing new asthma was 11.4% in the bottom quartile of EPA plus DHA intake and 6.6% in the top quartile. Cumulative exposure at 4 and 7 years (r=0.46) showed a stronger association: OR comparing those who had high intake at both time-points with those who had consistently low intake 0.33 (95% CI 0.15–0.70) in the GA/GG group and 1.19 (95% CI 0.52–2.71) in the AA group (p_interaction_=0.02).

**TABLE 3:** Odds ratio for incident asthma at 11 or 14 years according to intake of eicosapentaenoic acid (EPA) plus docosahexaenoic acid (DHA) from fish at 7 years of age, stratified by child's fatty acid desaturase (*FADS*) genotype in the Avon Longitudinal Study of Parents and Children

	**Quartiles of EPA plus DHA intake from fish**				**p_trend_- value^#^**	**p_interaction_- value**
	**Quartile 1**	**Quartile 2**	**Quartile 3**	**Quartile 4**		
**Median (IQR) intake mg·day^−1^**	5.51 (0.00–6.67)	22.1 (22.1–22.1)	41.9 (32.2–48.7)	94.0 (78.9–141)		
**Cases/non-cases**	100/950	81/947	112/1213	97/1043		
Model 1	1.00	0.80 (0.59–1.09)	0.86 (0.65–1.14)	0.86 (0.64–1.15)	0.56	
Model 2	1.00	0.80 (0.59–1.09)	0.85 (0.64–1.14)	0.86 (0.63–1.16)	0.55	
Model 3	1.00	0.82 (0.60–1.11)	0.87 (0.65–1.16)	0.86 (0.64–1.17)	0.56	
***FADS* genotype (rs1535): AA**						
Cases/non-cases	26/330	28/323	48/424	43/386		
Model 3	1.00	1.10 (0.62–1.95)	1.51 (0.90–2.55)	1.43 (0.83–2.46)	0.19	
***FADS* genotype (rs1535): GA/GG**						
Cases/non-cases	54/419	39/425	43/563	32/450		
Model 3	1.00	0.71 (0.45–1.10)	0.54 (0.35–0.83)	0.49 (0.31–0.79)	0.006	0.006

Data are presented as n/n or OR (95% CI), unless otherwise stated. IQR: interquartile range. **^#^**: linear trend was tested by treating the median values of quartiles as a continuous variable. Multivariable model 1: sex and total energy intake at 7 years. Multivariable model 2: further adjusted for maternal education, housing tenure during pregnancy, financial difficulty during pregnancy and maternal ethnicity. Multivariable model 3: further adjusted for maternal history of atopic disease, maternal age at delivery, exclusive breastfeeding, childcare by day nursery at 15 months of age, maternal smoking, older sibling, younger sibling and season when the food frequency questionnaire was completed.

Intakes of total *n*-6 or arachidonic acid were not associated with incident asthma, either overall or when stratified by *FADS* genotype (supplementary table E2). rs1535 was not associated with incident asthma (OR per minor G allele 0.91, 95% CI 0.76–1.09). We observed no evidence of associations between intakes of fish or EPA plus DHA from fish at 7 years and 547 (12.7%) cases of incident eczema or 933 (19.6%) cases of incident hay fever at 11 or 14 years, either overall or when stratified by *FADS* genotype (data not shown).

### Sensitivity analyses

**FIGURE 2:**
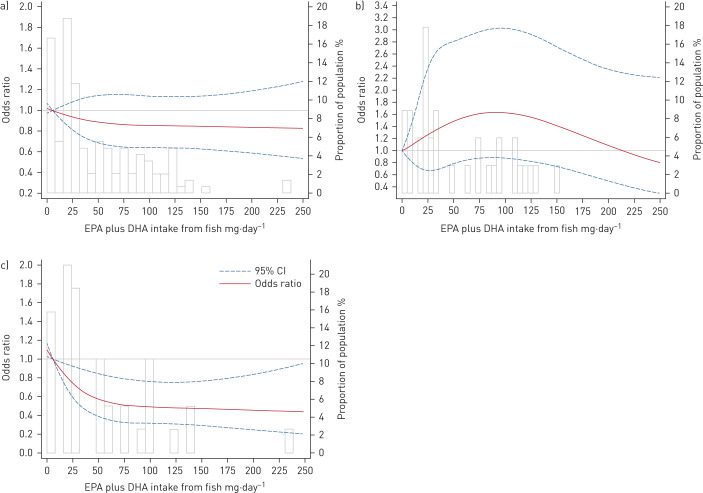
Dose–response relationship between eicosapentaenoic acid (EPA) plus docosahexaenoic acid (DHA) from fish and risk of incident asthma a) overall, b) in those homozygous for the major A allele and c) in carriers of the minor G allele using restricted cubic spline analysis in the Avon Longitudinal Study of Parents and Children. The model was adjusted for sex, total energy intake at 7 years, maternal education, housing tenure during pregnancy, financial difficulty during pregnancy, maternal ethnicity, maternal history of atopic disease, maternal age at delivery, exclusive breastfeeding, childcare by day nursery at 15 months of age, maternal smoking, older sibling, younger sibling and season when the food frequency questionnaire was completed. 56.3% of children were in the rs1535 GA/GG genotype group. The range of EPA plus DHA intake from fish was 0–675 mg·day^−1^ with skewness, so this was truncated for presentation purposes.

Associations with incident asthma, especially those among *FADS* minor allele carriers, did not materially change with further adjustment for age at first exposure to fish, health-conscious dietary pattern score, any supplement use, body mass index at 7 or 14 years, or genetic markers of population substructure derived by principal component analysis, nor after exclusion of 59 (1.3%) children of non-White mothers, 749 (16.5%) children with a history of food allergy, 390 (8.6%) children with extreme energy intakes, 98 (2.2%) children with wheeze at 7 years and 16 (0.3%) users of fish liver oil or *n*-3 supplements (supplementary table E3). The same pattern of associations was observed with EPA and DHA intakes separately (supplementary tables E4 and E5). Restricted cubic spline analysis showed a nonlinear association in carriers of the minor G allele (p_nonlinearity_=0.04), but not in the AA group (p_nonlinearity_=0.11) or overall (p_nonlinearity_=0.50) (figure 2). Furthermore, the main findings did not materially change when we used inverse probability weighting or energy-adjusted EPA and DHA intakes by the residual method (supplementary table E6).

Maternal intake of EPA plus DHA from fish during pregnancy was weakly associated with a lower risk of incident asthma at 11 or 14 years (adjusted OR comparing top quartile *versus* bottom quartile 0.69, 95% CI 0.48–0.98); however, there was no evidence of effect modification by *FADS* genotype (supplementary table E7). Importantly, the association between child's intake of EPA plus DHA at 7 years and incident asthma was independent of maternal intake in pregnancy (supplementary table E5).

Finally, we tested all other SNPs as a *post hoc* analysis and found 12 SNPs in strong linkage disequilibrium (10 SNPs with rs1535 and two SNPs with rs3734398), thus yielding identical findings (data not shown). We did not find evidence of significant effect modification by the other seven SNPs, although in line with rs1535 there was weak evidence of an inverse association between intake of EPA plus DHA from fish and incident asthma in carriers of the minor allele for some SNPs (supplementary table E8).

### Replication analyses

The characteristics of BAMSE study participants (n=2138) are summarised in supplementary table E9. Total intake of fish was lower in BAMSE children compared with ALSPAC children, but EPA and DHA intakes from fish were substantially higher in BAMSE as a result of higher oily fish intake (supplementary table E10). In the BAMSE cohort we sought to replicate the *n*-3 VLC-PUFA–*FADS* interaction on incident asthma, and confirmed that the association between mid-childhood intake of EPA plus DHA and incident asthma was modified by *FADS* genotype (rs1535), with similar effect estimates to those seen in ALSPAC, among 1187 (62.0%) carriers of the minor G allele (p_interaction_=0.03) (table 4). Similar interactions were also confirmed when we analysed intakes of EPA and DHA separately (supplementary table E11).

**TABLE 4:** Odds ratio for incident asthma at 12 or 16 years of age, according to intake of eicosapentaenoic acid (EPA) plus docosahexaenoic acid (DHA) from fish at 8 years of age, stratified by fatty acid desaturase (*FADS*) genotype in BAMSE (replication study)

	**Quartiles of EPA plus DHA intake from fish**				**p_trend_- value^#^**	**p_interaction_- value**
	**Quartile 1**	**Quartile 2**	**Quartile 3**	**Quartile 4**		
**Median (IQR) intake mg·day^−1^**	33.7 (18.5–47.4)	90.5 (70.5–117)	178 (158–197)	291 (248–362)		
**Cases/non-cases**	45/476	39/486	33/514	33/511		
Model 1	1.00	0.85 (0.54–1.33)	0.67 (0.42–1.07)	0.67 (0.42–1.07)	0.07	
Model 2	1.00	0.85 (0.54–1.33)	0.66 (0.41–1.07)	0.61 (0.38–1.00)	0.04	
Model 3	1.00	0.90 (0.57–1.41)	0.65 (0.40–1.06)	0.58 (0.35–0.96)	0.02	
***FADS* genotype (rs1535): AA**						
Cases/non-cases	16/166	14/174	13/164	15/164		
Model 3	1.00	0.88 (0.41–1.92)	0.75 (0.33–1.70)	0.84 (0.37–1.88)	0.64	
***FADS* genotype (rs1535): GA/GG**						
Cases/non-cases	24/257	22/268	20/298	15/283		
Model 3	1.00	0.96 (0.51–1.79)	0.72 (0.37–1.37)	0.52 (0.25–1.07)	0.05	0.03

Data are presented as n/n or OR (95% CI), unless otherwise stated. BAMSE: Swedish abbreviation for Children, Allergy, Milieu, Stockholm, Epidemiology; IQR: interquartile range. **^#^**: linear trend was tested by treating the median values of quartiles as a continuous variable. Multivariable model 1: sex and total energy intake at 8 years. Multivariable model 2: further adjusted for maternal education, parental occupation and maternal ethnicity. Multivariable model 3: further adjusted for maternal history of atopic disease, maternal age at delivery, exclusive breastfeeding, childcare by day nursery at 2 years of age, maternal smoking, older sibling and season when the food frequency questionnaire was completed.

## Discussion

In more than half of ALSPAC children who were carrying the minor G allele of a *FADS* polymorphism (rs1535), we found strong inverse associations between intake of fish, and EPA and DHA from fish, in mid-childhood and incident asthma. Replication of these gene–nutrient interactions on incident asthma in the BAMSE birth cohort confirmed that the main findings are unlikely to have arisen by chance. To the best of our knowledge, these are novel findings which were robust to various sensitivity analyses.

The overall relation between fish intake in mid-childhood and incident asthma has only been investigated in one previous study, namely the BAMSE cohort; in keeping with our findings, no association was observed in that study either [9]. However, we found weak evidence that, in carriers of the minor *FADS* allele, higher fish intake was associated with a lower risk of asthma, which has not been reported before. While fish intake during mid-childhood could potentially reflect similar familial dietary habits earlier in the life course, the findings of our study were unlikely to be confounded by maternal intake of *n*-3 VLC-PUFA from fish during pregnancy or by early exposure to fish in infancy.

The inverse association between fish intake and incident asthma in carriers of the minor G allele was largely explained by EPA and DHA. Longitudinal data on the link between dietary intake of EPA and DHA in childhood and incident asthma are lacking. While no previous study has reported effect modification of the association between dietary intake of *n*-3 VLC-PUFAs and asthma by *FADS* genotype in childhood, in a recent RCT by Bisgaard *et al.* [12] the protective effect of fish oil supplementation in pregnancy on risk of early childhood asthma was modified by the same *FADS* gene variant in mothers. Our findings extend those observations and suggest that there may be potential to prevent late childhood onset asthma in some individuals. In contrast to the ALSPAC cohort, the inverse associations seen in the BAMSE cohort as a whole may reflect the relatively higher *n*-3 VLC-PUFA intake and the higher *FADS* minor allele frequency (62% *versus* 56%).

We showed a stronger association when we compared consistently high *versus* consistently low intake at 4 and 7 years. This could reflect either the beneficial effect of more prolonged exposure or reduced exposure misclassification by using dietary data at two time-points. Nonetheless, it strengthens the case for a causal association. An important concern is potential reverse causation bias arising from disease-related modification of diet, especially in children with food allergy. However, our main findings did not materially change when we excluded children with any history of food allergy.

### Mechanisms

EPA and DHA can modulate inflammatory processes through various pathways, such as increasing mediators that are less pro-inflammatory, anti-inflammatory or inflammation resolving [4, 25]. While plasma concentrations of *n*-3 VLC-PUFAs at 8 years were inversely associated with incident asthma previously in BAMSE [26], use of biomarkers cannot differentiate between extrinsic (dietary) and intrinsic sources. The fish oil supplement RCT in pregnant women by Bisgaard *et al.* [12] also found effects only for offspring asthma and not for other allergy-related disorders, which suggests that the anti-inflammatory mechanisms may be confined to the airways. The main endogenous source of *n*-3 VLC-PUFAs is through a pathway mainly regulated by desaturase enzymes encoded by *FADS* genes, which converts the plant-derived *n*-3 PUFA precursor α-linolenic acid (ALA) to EPA and then DHA. Carriers of the minor allele of rs1535 (a representative SNP in *FADS2*) have a lower ALA-to-EPA conversion rate. They therefore tend to have lower blood concentrations of EPA and DHA [11], and are thus more dependent on dietary sources. This is likely to explain why a higher dietary intake of EPA and DHA was associated with a lower risk of incident asthma in this genetic subgroup. Of note, while *FADS2* influences both *n*-3 and *n*-6 PUFA pathways, the effect modification we observed was specific to intake of EPA and DHA, not *n*-6 fatty acids or arachidonic acid, further strengthening causal inference.

### Strengths and limitations

Strengths of the ALSPAC birth cohort include its population-based prospective design, large size, rich information on diet (at multiple time-points) and potential confounders, and availability of *FADS* genotype data. The detailed, repeated, phenotypic outcome measurements provided an opportunity to study incident rather than prevalent cases. As with any observational study, the possibility of unmeasured or residual confounding cannot be ruled out, although we controlled for numerous potential confounders in the analyses. We could not examine longitudinal associations with atopy and atopic asthma, because skin prick testing was only done at 7 years of age. A proportion of eligible children at 7 years (25.6%) were not included in our analyses due to lack of information on asthma status at any later time-point. However, loss-to-follow-up bias has been shown to only slightly modify associations in longitudinal studies [27] and our inverse probability weighting analysis confirmed that it is unlikely to have biased our results. Although the FFQ that we used had not been formally calibrated against other instruments such as diet diaries, it was based on the one used by Yarnell *et al*. [28] that has been validated against weighed dietary records, and updated in the light of a local weighed dietary survey [29]. While there is likely to have been some misclassification of the dietary exposures, especially as the FFQ lacked quantitative information on portion sizes, the interaction between *n*-3 VLC-PUFA intake and *FADS* genotype argues against substantial exposure misclassification. Furthermore, any such misclassification is likely to have been random with respect to asthma, which would tend to bias effect estimates towards the null. In this context, the replication of our findings in an independent cohort study (BAMSE) is strengthened by the fact that preferred fish species and preparation methods differ between the two countries. However, it would be premature to give clear recommendations regarding the absolute intake of *n*-3 VLC-PUFA needed to achieve maximum benefit in terms of asthma risk given the semiquantitative nature of the FFQs, the differences in estimated *n*-3 intake between the ALSPAC and BAMSE populations, and more importantly the inherent limitations of observational studies in establishing causality.

Current recommendations in the UK are to consume two servings of fish per week (equivalent to 280 g·week^−1^ for an adult), one of which should be of oily fish [30]. In the last decade, fish consumption in children has slightly increased in the UK [31]; however, only 4.2% of children are achieving the recommended intake [30]. If our findings are causal, this might ultimately lead to a strategy of personalised primary prevention in a large subgroup of the population, according to genotype. In the meantime, public health messages to increase intake of fish should be heeded.

### Conclusions

Although the evidence for an association overall was lacking in ALSPAC, we have replicated the finding that in children with a common *FADS* gene variant associated with poorer endogenous synthesis of *n*-3 VLC-PUFAs, a higher intake of EPA and DHA from fish in childhood was associated with a lower risk of incident asthma.

## Supplementary material

10.1183/13993003.03633-2020.Supp1 **Please note:** supplementary material is not edited by the Editorial Office, and is uploaded as it has been supplied by the author.
Supplementary material ERJ-03633-2020.SUPPLEMENT

## Shareable PDF

10.1183/13993003.03633-2020.Shareable1 This one-page PDF can be shared freely online.
Shareable PDF ERJ-03633-2020.Shareable
