# Supplementary material for: Intake of n-3 polyunsaturated fatty acids in childhood, FADS genotype and incident asthma
Source: Eur Respir J. 2021 Sep 2;58(3):2003633. doi: 10.1183/13993003.03633-2020 (PMC8411098; doi:10.1183/13993003.03633-2020)
Supplement: Supplementary file 1 [file ERJ-03633-2020.SUPPLEMENT.pdf]

Online Data Supplement

Intake of n-3 polyunsaturated fatty acids in childhood, FADS genotype, and incident asthma

Mohammad Talaei, Emmanouela Sdona, Philip C. Calder, Louise R. Jones, Pauline M. Emmett, Raquel Granell, Anna Bergström, Erik Melén, Seif O. Shaheen

Corresponding author:

Prof. Seif Shaheen, Institute of Population Health Sciences, Barts and The London School of Medicine and Dentistry, 58 Turner Street, London E1 2AB. Email: s.shaheen@qmul.ac.uk; Tel: +44 (0)20 7882 2480

Dr Mohammad Talaei, Institute of Population Health Sciences, Barts and The London School of Medicine and Dentistry, 58 Turner Street, London, E1 2AB, UK. E-mail: mohammad.talaei@u.nus.edu; Tel: +44(0)20 7882 2499

Contents

Further details (ALSPAC) **3**

Exposure assessment 3

Secondary outcomes 3

Information on covariates 3

Genotyping 4

List of n-3 SNPs 5

Multivariable models 6

Sensitivity analyses 6

Restricted cubic spline analysis 7

Inverse probability weighting 7

Further details (BAMSE) **8**

Study design 8

Exposure assessment 9

Outcome assessment 9

Statistical analysis 10

References **12**

Supplementary tables and figures **14**

Figure E1. Study profile (ALSPAC). 14

Figure E2: Odds ratio (95% confidence interval) for incident asthma at 11 or 14 years according to intake of EPA plus DHA from fish at 7 years of age, stratified by FADS genotype (rs1535), in ALSPAC 15

Table E1: Participant characteristics according to rs1535 genotype in ALSPAC 16

Table E2: Odds ratio (95% confidence interval) for incident asthma at 11 or 14 years according to intake of arachidonic acid and total n-6 fatty acids at 7 years of age, stratified by child's *FADS* genotype in ALSPAC 18

Table E3: Odds ratio (95% confidence interval) for incident asthma at 11 or 14 years according to intake of EPA plus DHA from fish at 7 years of age, stratified by child's *FADS* genotype in ALSPAC (sensitivity analyses) 19

Table E4: Odds ratio (95% confidence interval) for incident asthma at 11 or 14 years according to intake of EPA from fish at 7 years of age, stratified by child's *FADS* genotype in ALSPAC 21

Table E5: Odds ratio (95% confidence interval) for incident asthma at 11 or 14 years according to intake of DHA from fish at 7 years of age, stratified by child's *FADS* genotype in ALSPAC 22

Table E6: Odds ratio (95% confidence interval) for incident asthma at 11 or 14 years according to intake of EPA plus DHA from fish at 7 years of age adjusted for total energy intake and applying inverse probability weighting, stratified by child's *FADS* genotype in ALSPAC 23

Table E7: Odds ratio (95% confidence interval) for incident asthma at 11 or 14 years according to maternal intake of EPA plus DHA from fish at 32 weeks of gestation, stratified by maternal *FADS* genotype in ALSPAC 24

Table E8: Odds ratio (95% confidence interval) for incident asthma at 11 or 14 years according to intake of EPA plus DHA from fish at 7 years of age, stratified by other child polymorphisms related to n-3 fatty acid metabolism (those not in linkage disequilibrium †) in ALSPAC 25

Table E9. Participant characteristics in the BAMSE cohort 27

Table E10. Intakes of fish, and EPA and DHA from fish, in BAMSE and ALSPAC. 28

Table E11: Odds ratio (95% confidence interval) for incident asthma at 12 or 16 years of age, according to intake of EPA and DHA from fish at 8 years of age, stratified by *FADS* genotype in BAMSE 29

Further details (ALSPAC)

Exposure assessment

Fish intake was covered by five items: shellfish (prawns, crab, cockles, mussels), white fish in breadcrumbs or batter (e.g. fish fingers/shapes, chip shop fish, breaded cod), white fish without coating (e.g. grilled fish, cod in parsley sauce), tuna, and other fish (pilchards, sardines, mackerel, herring, kippers, trout, salmon). Standard portion sizes based on typical consumption patterns in Britain [1] were adapted for the age of children and used to estimate the daily intake of each food group.

Secondary outcomes

Current eczema and hay fever in children at ages 7.5, 11, and 14 years were defined by a positive answer to the question “Has your child had any of the following in the past 12 months" that included eczema and hay fever items. Among those children who were without eczema or hay fever at 7.5 years, we defined incident cases if mothers reported these conditions at 11 or 14 years. Wheeze at 42 months (3.5y) and 91 months (~7.5y) were defined as present if the response to any of the two questions asking about wheezing in the past 12 months was “yes” at each time point. Together with prevalent current doctor-diagnosed asthma at 7 years, presence of wheezing at 3.5 years and 7 years were analysed in relation to maternal n-3 intake during pregnancy. As data on asthma diagnosis were not available for ages below 7 years, we instead considered incident wheeze at 7 years, defined as presence of wheezing at 7 years among those with no report of wheezing at 3.5 years of age. This was analysed in relation to dietary n-3 intake at 4 years of age.

Information on covariates

A maternal history of hay fever, asthma, and eczema was ascertained at 12 weeks of gestation, and any positive response was considered as a maternal history of atopic disease. When the child was 6 months and 15 months old, mothers were asked at what age the child had started eating fish. We defined ‘early fish exposure’ in three categories: before 6 months, 6-9 months, and after 9 months. Mothers were asked

how many cigarettes they smoked per day when the child was 7 years of age. We defined childhood food allergy if there was any such report by mothers at 6 (to milk), 30, 54, or 81 months of age.

Data on maternal ethnicity and indicators of socioeconomic status (maternal education, housing tenure and financial difficulty in pregnancy) were collected at various time points during pregnancy (8, 18, and 32 weeks of gestation) and at 8 weeks postpartum. Maternal age was recorded at delivery. Data on breast-feeding by the 3rd month, and childcare by day nursery, were collected at 6 and 15 months of age, respectively. Number of older and younger siblings was asked at 7 years; if data were missing we used data on parity to calculate the number of older siblings. Child's body mass index was calculated as weight (kg) divided by height squared (m^2), measured at ages 7 and 14 years. A health-conscious dietary pattern was previously defined using principal component analysis and was associated with better nutrient profiles than the processed patterns, which tended to be energy-dense and nutrient-poor [2].

Genotyping

The majority of the children's DNA samples were extracted from cord blood or venous blood collected at age 7 years, with a small number extracted from venous blood collected at 43–61 months. ALSPAC children were genotyped using the Illumina HumanHap550 quad chip genotyping platforms by 23andme subcontracting the Wellcome Trust Sanger Institute, Cambridge, UK and the Laboratory Corporation of America, Burlington, NC, US. The resulting raw genome-wide data were subjected to standard quality control methods. Individuals were excluded on the basis of gender mismatches; minimal or excessive heterozygosity; disproportionate levels of individual missingness (>3%) and insufficient sample replication (IBD < 0.8). Population stratification was assessed by multidimensional scaling analysis and compared with Hapmap II (release 22) European descent (CEU), Han Chinese, Japanese and Yoruba reference populations; all individuals with non-European ancestry were removed. SNPs with a minor allele frequency of < 1%, a call rate of < 95% or evidence for violations of Hardy-Weinberg equilibrium (P < 5E-7) were removed. Cryptic relatedness was measured as proportion of identity by descent (IBD > 0.1). Related subjects that passed all other quality control thresholds were retained during subsequent phasing and imputation. 9,115 subjects and 500,527 SNPs passed these quality control filters.
ALSPAC mothers were genotyped using the Illumina human660W-quad array at Centre National de

Génotypage (CNG) and genotypes were called with Illumina GenomeStudio. PLINK (v1.07) was used to carry out quality control measures on an initial set of 10,015 subjects and 557,124 directly genotyped SNPs. SNPs were removed if they displayed more than 5% missingness or a Hardy-Weinberg equilibrium P value of less than 1.0e-06. Additionally SNPs with a minor allele frequency of less than 1% were removed. Samples were excluded if they displayed more than 5% missingness, had indeterminate X chromosome heterozygosity or extreme autosomal heterozygosity. Samples showing evidence of population stratification were identified by multidimensional scaling of genome-wide identity by state pairwise distances using the four HapMap populations as a reference, and then excluded. Cryptic relatedness was assessed using a IBD estimate of more than 0.125 which is expected to correspond to roughly 12.5% alleles shared IBD or a relatedness at the first cousin level. Related subjects that passed all other quality control thresholds were retained during subsequent phasing and imputation. 9,048 subjects and 526,688 SNPs passed these quality control filters.

We combined 477,482 SNP genotypes in common between the sample of mothers and sample of children. We removed SNPs with genotype missingness above 1% due to poor quality (11,396 SNPs removed) and removed a further 321 subjects due to potential ID mismatches. This resulted in a dataset of 17,842 subjects containing 6,305 duos and 465,740 SNPs (112 were removed during liftover and 234 were out of HWE after combination). We estimated haplotypes using ShapeIT (v2.r644) which utilises relatedness during phasing. We obtained a phased version of the 1000 genomes reference panel (Phase 1, Version 3) from the Impute2 reference data repository (phased using ShapeIt v2.r644, haplotype release date Dec 2013). Imputation of the target data was performed using Impute V2.2.2 against the reference panel (all polymorphic SNPs excluding singletons), using all 2186 reference haplotypes (including non-Europeans).

List of n-3 SNPs

Through literature review we found 20 SNPs that were involved in the metabolism of n-3 fatty acids, or were correlated with serum concentration of EPA and/or DHA: rs174547 [3, 4], rs780094 [3], rs3734398 [3], rs12662634 [3], rs3798713 [3], rs174538 [3], rs174535 [3], rs2236212 [3], rs1535 [3, 5, 6], rs174575

[5-8], rs174448 [5], rs11693320 [9], rs174602 [10], rs174556 [7, 8, 11], rs174450 [12], rs174537 [13, 14], rs174576 [13], rs174545 [7, 8, 14], rs174583 [14], rs174561 [7, 8, 14].

Multivariable models

In the multivariable models, we first adjusted for sex and total energy intake (kJ·day−1) at 7 years. The second model additionally included maternal ethnicity (white, non-white) and three indicators of socioeconomic status, namely, maternal education (secondary education, vocational, O level, A level, degree, and missing), housing tenure during pregnancy (mortgaged/owned, council rented, non-council rented, unknown/missing), and financial difficulty during pregnancy (yes/no). In the third model, we further adjusted for maternal history of atopic disease (yes/no), maternal age at delivery (continuous), breastfeeding by the 3rd month (never, stopped/non-exclusive, exclusive), childcare by day nursery at 15 months of age (yes/no), maternal smoking when the child was 7 years of age (none, 1-9, 10-19, and ≥20/day), older sibling (yes/no), younger sibling (yes/no), and season when the FFQ was completed (winter, spring, summer, autumn). Data on potential confounders in multivariable models were missing for 4.2% at most and included in the analyses as separate 'missing' categories.

If evidence for associations with fish intake persisted after adjustment for all potential confounders, we conducted additional adjustment for child's BMI (continuous) and intakes of EPA and DHA from fish, vitamin D, and selenium (quartiles) as potential mediators. When EPA and DHA intakes were the exposures of interest, we further adjusted for dietary intake of vitamin D and selenium as potential confounders.

Sensitivity analyses

We explored the impact of excluding children of non-white mothers, those children with any history of food allergy before 7 years of age, wheeze at 7 years, an extreme total energy intake above the 95th percentile or below the 5th percentile, and those who consumed fish liver oil or omega-3 supplements. We also used the residual method [15] to adjust dietary EPA and DHA for total energy intake and examined the new adjusted variables in the same multivariable models for original ones. The sensitivity of our findings to adjustment for other potential confounders was tested by further adjusting for maternal intake

of n−3 PUFA from fish at 32 weeks of gestation (quartiles), age at first exposure to fish (<6m, 6-<9m, and ≥9m), BMI (continuous), any supplement use (as an indicator of health related behaviours), and health-conscious dietary pattern at 7 years (quartiles, as an indicator of a generally healthy diet) [2]. To address possible residual confounding by population substructure (ancestral differences), we also adjusted for the first 10 principal components (PC) derived by PC analysis from ALSPAC genome-wide data.

Finally, we explored 19 other SNPs involved in n-3 PUFA metabolism and checked for linkage disequilibrium (LD) with our main SNP using LDlink (https://ldlink.nci.nih.gov/). We considered a SNP to be in LD with rs1535 if R^2 >0.90 (9 SNPs) or D´=1 (rs174575). As a *post hoc* analysis, we tested the interaction between EPA plus DHA intake from fish and 7 SNPs not in LD with each other on the risk of incident asthma at 11 or 14 years.

Restricted cubic spline analysis

Restricted cubic spline analysis was used to examine the shape of relationship between EPA plus DHA and incident asthma in multivariable-adjusted models overall and stratified by FADS rs1535. We selected the number of knots based on the values of Akaike information criteria (AIC) to fit the best-approximating model, chose the first knot as reference, and tested for linearity by the Wald-test. Accordingly, the lowest AIC (best fitted model) was obtained by 4 knots in the AA group and by 3 knots in the GA/GG group and overall.

Inverse probability weighting

Inverse probability weighting is a technique to correct for selection bias [16]. In a two-step method, the probability of selection in the study is estimated for everyone based on a given set of covariates and exposure; then the inverse of this probability is included in the analysis as a weight. Inverse probability weighting creates a pseudo-population in which each selected subject accounts for those with similar characteristics who were not selected.

Accordingly, among 7,188 children with data on fish intake who were not diagnosed with current asthma at 7 years, we estimated the probability of selection of 4,543 children for given values of covariates using a logistic regression model. Unselected children were those of unknown asthma status at 7, 11, or 14 years. These covariates included all factors in model 3 (namely, sex, total energy intake, maternal education, housing tenure during pregnancy, financial difficulty during pregnancy, maternal ethnicity, maternal history of atopic disease, maternal age, breastfeeding, childcare, maternal smoking, older sibling, younger sibling, and season of dietary data collection), plus quartiles of fish intake, quartiles of health-conscious dietary pattern score, and history of food allergy. Then, we assigned the inverse of this probability as the weight for each participant, and carried out a multivariable weighted logistic regression analysis to test the associations of fish and n-3 LCPUFA intake with incident asthma in a pseudo-population, which, in contrast to the selected population, is unaffected by selection bias due to these factors. In other words, this approach tests if the observed associations in the main analysis were sensitive to unknown asthma status at baseline or loss to follow-up.

Further details (BAMSE)

Study design

The replication study was conducted within the population-based birth cohort BAMSE (Swedish abbreviation for Children, Allergy, Milieu, Stockholm, Epidemiology), to which 4,089 children (born 1994-1996, comprising 75% of all eligible children) from predefined areas of Stockholm County, Sweden, have been followed repeatedly from infancy [17, 18]. In brief, baseline information was collected through parental questionnaires when the children were on average two months old, and follow-up questionnaires eliciting information on symptoms of allergic diseases and selected exposures were answered by the parents when the children were 1, 2, 4, 8 and 12 years, and by the adolescents themselves at 16 years. The response rates were 96%, 94%, 91%, 84%, 82% and 78%, respectively. At ages 8 and 16 years, participants were invited to clinical examinations, which included anthropometric measurements, lung function testing and blood sampling using standardized methods. Blood samples

have been analyzed for specific IgE to common inhalant and food allergens, as well as used for DNA extraction. The study was approved by the Ethics Committee of Karolinska Institutet, Stockholm, Sweden, and written informed consent was obtained from parents at 8 and 12 years and study participants at 16 years.

Exposure assessment

At the 8-year-clinical examination, parents (together with their child) were asked to fill in a food frequency questionnaire (FFQ) containing questions about 98 foods and beverages frequently consumed in Sweden, including six questions on fish intake [herring/mackerel and salmon fishes (categorized as oily fish), as well as codfish/pollock/pike, fish fingers, tuna fish and seafood (shrimp, crayfish, crab)]. Most often the FFQ was filled out by a parent (57%) or by a parent together with the child (40%). Children (n = 2,614) were asked how often, on average, they had consumed each type of food or beverage during the past 12 months. There were 10 pre-specified response categories ranging from 'never' to '≥3 times/day' [19].

Intakes of dietary polyunsaturated fatty acids (PUFAs) were computed from the FFQ by multiplying the frequency of consumption of each food item by its nutrient content per serving, using composition values obtained from the Swedish National Food Administration Database and summarized over foods and beverages [20]. Nutrient intakes were adjusted for total energy intake by using the residuals method [15]. Daily intakes of omega-3 (n−3) PUFAs (mg/day) from fish only, including their long chain subtypes (LC-PUFA), eicosapentaenoic acid (EPA) and docosahexaenoic acid (DHA), were calculated.

Outcome assessment

At 8, 12 and 16 years, we defined current doctor-diagnosed asthma if mothers responded positively to the question "Has a doctor ever actually said that your study child has asthma?", and to at least one of the concurrent questions which asked if, in the last 12 months, the child had had at least one episode of wheeze, and used asthma medication. Among those children who were not identified as having current doctor-diagnosed asthma at 8 years, we defined those with current doctor-diagnosed asthma at 12 or 16 years as cases of incident asthma.

At 8, 12 and 16 years, we defined eczema if there was a positive reply for eczema symptoms (dry skin in combination with itchy rash and typical localisation) in the last 12 months. Among those children who were not identified as having eczema at 8 years, we defined those with eczema at 12 or 16 years as cases of incident eczema.

Statistical analysis

Among 2,614 children with dietary data available at 8 years, data were complete on incident asthma for 2,159. Participants with baseline questionnaire data, data on total fish and nutrient intake at age 8 years, and complete data on incident asthma, were included in the present study (n=2,138, 52% of the original cohort).

We employed logistic regression to examine associations of n−3 PUFA from fish with incident asthma using the lowest quartile of intake as the reference category. Additionally, we examined associations of fish intake and n−3 PUFA from fish with incident eczema. Linear trend was tested by including median intake of quartiles as a pseudo-continuous variable in the models.

In the multivariable models, we first adjusted for sex and total energy intake (kcal·day−1) at 8 years. The second model additionally included indicators of socioeconomic status, namely, maternal education (9-year compulsory school, 2-year secondary school, 3-4-year secondary school, university or college), parental occupation (blue collar, lower white collar, higher white collar, other), and maternal ethnicity (European, non-European). In the third model, we further adjusted for maternal history of atopic disease (yes/no), maternal age at delivery (continuous), exclusive breastfeeding (less than four months, four months or more), childcare by day nursery at 2 years of age (yes/no), maternal smoking when the child was 8 years of age (none, 1-9, 10-19, and ≥20/day), older sibling (yes/no) and season when the FFQ was completed (winter, spring, summer, autumn).

Fatty acid desaturase (*FADS*) single nucleotide polymorphism (SNP, rs1535) was available from GWAS data in BAMSE for a total of 2,712 subjects (i.e. 66% of the original cohort). The first round of genotyping (Illumina 610k) was done using DNA from 485 asthma cases and controls from the follow-up at eight years of age [21]. Recently, a total of 2,378 16-year-old children were additionaly genotyped with

the Ilumina Infinium Global Screening Array-24.10 BeadChip following the same sample collection procedures, protocols and questionnaires. Quality control (QC) was performed following the Ricopili pipeline [22]. Data were imputed using the Haplotype Reference Consortium 1.1 reference panel [23] with a pre-phasing step using Eagle2. Variants with imputation quality (Rsq) ≥ 0.3 were retained. After QC, a total of 448 (from the eight-year follow-up) and 2,264 subjects (from the 16-year follow-up) with phenotype data available were retained in the genetic analyses for BAMSE.

We carried out stratified analyses, *a priori*, to explore potential modifications of dietary associations by maternal history of atopy, and *FADS* genotype (rs1535 major A allele homozygous vs. heterozygotes plus homozygous for minor G allele, combined). Potential interactions were assessed by testing cross-product terms of these three factors with median values of dietary quartiles as a continuous factor in regression models. Maternal history of atopy was defined as a mother with doctor diagnosed asthma and asthma medication, or doctor diagnosed hay fever, or doctor diagnosed eczema at baseline.

References

1. Ministry of Agriculture Fisheries and Food. Food Portion Sizes, London, HMSO 1991.

2. Emmett PM, Jones LR, Northstone K. Dietary patterns in the Avon Longitudinal Study of Parents and Children. *Nutr Rev* 2015: 73 Suppl 3: 207-230.

3. Lemaitre RN, Tanaka T, Tang W, Manichaikul A, Foy M, Kabagambe EK, Nettleton JA, King IB, Weng LC, Bhattacharya S, Bandinelli S, Bis JC, Rich SS, Jacobs DR, Jr., Cherubini A, McKnight B, Liang S, Gu X, Rice K, Laurie CC, Lumley T, Browning BL, Psaty BM, Chen YD, Friedlander Y, Djousse L, Wu JH, Siscovick DS, Uitterlinden AG, Arnett DK, Ferrucci L, Fornage M, Tsai MY, Mozaffarian D, Steffen LM. Genetic loci associated with plasma phospholipid n-3 fatty acids: a meta-analysis of genome-wide association studies from the CHARGE Consortium. *PLoS Genet* 2011: 7(7): e1002193.

4. Liu F, Li Z, Lv X, Ma J. Dietary n-3 polyunsaturated fatty acid intakes modify the effect of genetic variation in fatty acid desaturase 1 on coronary artery disease. *PLoS One* 2015: 10(4): e0121255.

5. Harslof LB, Larsen LH, Ritz C, Hellgren LI, Michaelsen KF, Vogel U, Lauritzen L. FADS genotype and diet are important determinants of DHA status: a cross-sectional study in Danish infants. *Am J Clin Nutr* 2013: 97(6): 1403-1410.

6. Steer CD, Hibbeln JR, Golding J, Davey Smith G. Polyunsaturated fatty acid levels in blood during pregnancy, at birth and at 7 years: their associations with two common FADS2 polymorphisms. *Hum Mol Genet* 2012: 21(7): 1504-1512.

7. Standl M, Sausenthaler S, Lattka E, Koletzko S, Bauer CP, Wichmann HE, von Berg A, Berdel D, Kramer U, Schaaf B, Lehmann I, Herbarth O, Klopp N, Koletzko B, Heinrich J, Giniplus, Group LIS. FADS gene cluster modulates the effect of breastfeeding on asthma. Results from the GINIplus and LISAplus studies. *Allergy* 2012: 67(1): 83-90.

8. Standl M, Sausenthaler S, Lattka E, Koletzko S, Bauer CP, Wichmann HE, von Berg A, Berdel D, Kramer U, Schaaf B, Roder S, Herbarth O, Klopp N, Koletzko B, Heinrich J, Giniplus, Group LIS. FADS gene variants modulate the effect of dietary fatty acid intake on allergic diseases in children. *Clin Exp Allergy* 2011: 41(12): 1757-1766.

9. Xu J, Gaddis NC, Bartz TM, Hou R, Manichaikul AW, Pankratz N, Smith AV, Sun F, Terzikhan N, Markunas CA, Patchen BK, Schu M, Beydoun MA, Brusselle GG, Eiriksdottir G, Zhou X, Wood AC, Graff M, Harris TB, Ikram MA, Jacobs DR, Jr., Launer LJ, Lemaitre RN, O'Connor GT, Oelsner EC, Psaty BM, Vasan RS, Rohde RR, Rich SS, Rotter JI, Seshadri S, Smith LJ, Tiemeier H, Tsai MY, Uitterlinden AG, Voruganti VS, Xu H, Zilhao NR, Fornage M, Zillikens MC, London SJ, Barr RG, Dupuis J, Gharib SA, Gudnason V, Lahousse L, North KE, Steffen LM, Cassano PA, Hancock DB. Omega-3 Fatty Acids and Genome-Wide Interaction Analyses Reveal DPP10-Pulmonary Function Association. *Am J Respir Crit Care Med* 2019: 199(5): 631-642.

10. Gonzalez-Casanova I, Rzehak P, Stein AD, Garcia Feregrino R, Rivera Dommarco JA, Barraza-Villarreal A, Demmelmair H, Romieu I, Villalpando S, Martorell R, Koletzko B, Ramakrishnan U. Maternal single nucleotide polymorphisms in the fatty acid desaturase 1 and 2 coding regions modify the impact of prenatal supplementation with DHA on birth weight. *Am J Clin Nutr* 2016: 103(4): 1171-1178.

11. Molto-Puigmarti C, van Dongen MC, Dagnelie PC, Plat J, Mensink RP, Tan FE, Heinrich J, Thijs C. Maternal but not fetal FADS gene variants modify the association between maternal long-chain PUFA intake in pregnancy and birth weight. *J Nutr* 2014: 144(9): 1430-1437.

12. Bernard JY, Pan H, Aris IM, Moreno-Betancur M, Soh SE, Yap F, Tan KH, Shek LP, Chong YS, Gluckman PD, Calder PC, Godfrey KM, Chong MF, Kramer MS, Karnani N, Lee YS. Long-chain polyunsaturated fatty acids, gestation duration, and birth size: a Mendelian randomization study using fatty acid desaturase variants. *Am J Clin Nutr* 2018: 108(1): 92-100.

13. Roke K, Mutch DM. The role of FADS1/2 polymorphisms on cardiometabolic markers and fatty acid profiles in young adults consuming fish oil supplements. *Nutrients* 2014: 6(6): 2290-2304.

14. Gillingham LG, Harding SV, Rideout TC, Yurkova N, Cunnane SC, Eck PK, Jones PJ. Dietary oils and FADS1-FADS2 genetic variants modulate [13C]alpha-linolenic acid metabolism and plasma fatty acid composition. *Am J Clin Nutr* 2013: 97(1): 195-207.

15. Willett WC, Howe GR, Kushi LH. Adjustment for total energy intake in epidemiologic studies. *Am J Clin Nutr* 1997: 65(4 Suppl): 1220S-1228S; discussion 1229S-1231S.

16. Hernan MA, Hernandez-Diaz S, Robins JM. A structural approach to selection bias. *Epidemiology* 2004: 15(5): 615-625.

17. Ekstrom S, Magnusson J, Kull I, Lind T, Almqvist C, Melen E, Bergstrom A. Maternal body mass index in early pregnancy and offspring asthma, rhinitis and eczema up to 16 years of age. *Clin Exp Allergy* 2015: 45(1): 283-291.

18. Wickman M, Kull I, Pershagen G, Nordvall SL. The BAMSE project: presentation of a prospective longitudinal birth cohort study. *Pediatr Allergy Immunol* 2002: 13(s15): 11-13.

19. Magnusson J, Kull I, Westman M, Hakansson N, Wolk A, Melen E, Wickman M, Bergstrom A. Fish and polyunsaturated fat intake and development of allergic and nonallergic rhinitis. *J Allergy Clin Immunol* 2015: 136(5): 1247-1253 e1241-1242.

20. Bergström L, Kylberg E, Hagman U, Erikson H, Bruce Å. The food composition database KOST: the National Food Administration’s information system for nutritive values of food. *Vår föda* 1991: 43: 439-447.

21. Moffatt M, Gut I, Demenais F, Strachan D, Bouzigon E, Heath S, von Mutius E, Farrall M, Lathrop M, Cookson W, Consortium. G. A large-scale, consortium-based genomewide association study of asthma. *N Engl J Med* 2010: 363(13): 1211-1221.

22. Lam M, Awasthi S, Watson H, Goldstein J, Panagiotaropoulou G, Trubetskoy V, Karlsson R, Frei O, Fan C, De Witte W, Mota N, Mullins N, K. B, Lee H, Wray N, Skarabis N, Huang H, Neale B, Daly M, Mattheissen M, Walters R, Ripke S. RICOPILI: Rapid Imputation for COnsortias PIpeLIne. *Bioinformatics* 2019.

23. McCarthy S, Das S, Kretzschmar W, Delaneau O, Wood A, Teumer A, Kang H, Fuchsberger C, Danecek P, Sharp K, Luo Y, Sidore C, Kwong A, Timpson N, Koskinen S, Vrieze S, Scott L, Zhang H, Mahajan A, Veldink J, Peters U, Pato C, van Duijn C, Gillies C, Gandin I, Mezzavilla M, Gilly A, Cocca M, Traglia M, Angius A, Barrett J, Boomsma D, Branham K, Breen G, Brummett C, Busonero F, Campbell H, Chan A, Chen S, Chew E, Collins F, Corbin L, Smith G, Dedoussis G, Dorr M, Farmaki A, Ferrucci L, Forer L, Fraser R, Gabriel S, Levy S, Groop L, Harrison T, Hattersley A, Holmen O, Hveem K, Kretzler M, Lee J, McGue M, Meitinger T, Melzer D, Min J, Mohlke K, Vincent J, Nauck M, Nickerson D, Palotie A, Pato M, Pirastu N, McInnis M, Richards J, Sala C, Salomaa V, Schlessinger D, Schoenherr S, Slagboom P, Small K, Spector T, Stambolian D, Tuke M, Tuomilehto J, Van den Berg L, Van Rheenen W, Volker U, Wijmenga C, Toniolo D, Zeggini E, Gasparini P, Sampson M, Wilson J, Frayling T, de Bakker P, Swertz M, McCarroll S, Kooperberg C, Dekker A, Altshuler D, Willer C, Iacono W, Ripatti S, Soranzo N, Walter K, Swaroop A, Cucca F, Anderson C, Myers R, Boehnke M, McCarthy M, Durbin R, Consortium. HR. A reference panel of 64,976 haplotypes for genotype imputation. *Nat Genet* 2016: 48(10): 1279-1283.

Supplementary tables and figures

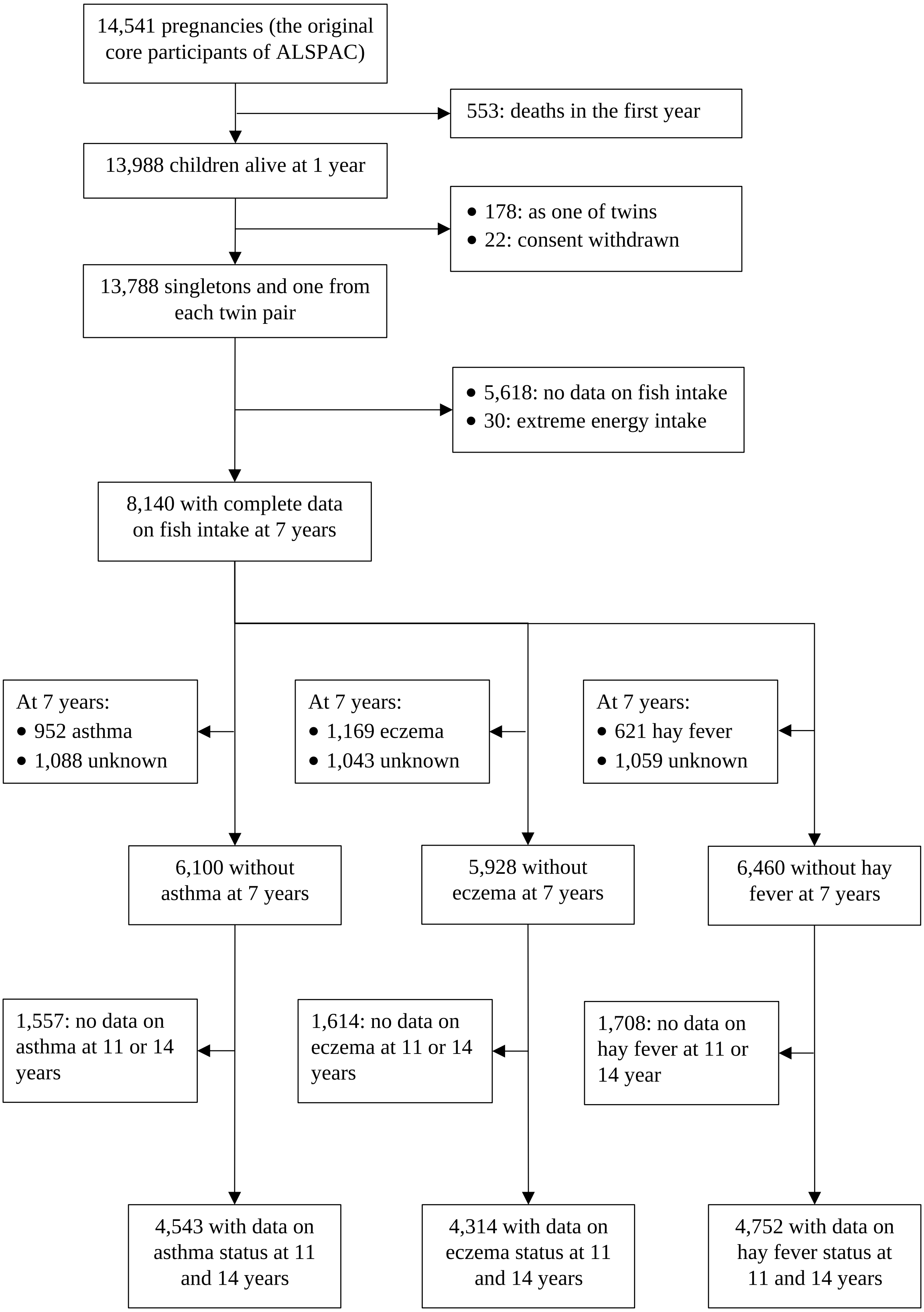

Figure E1. Study profile (ALSPAC).

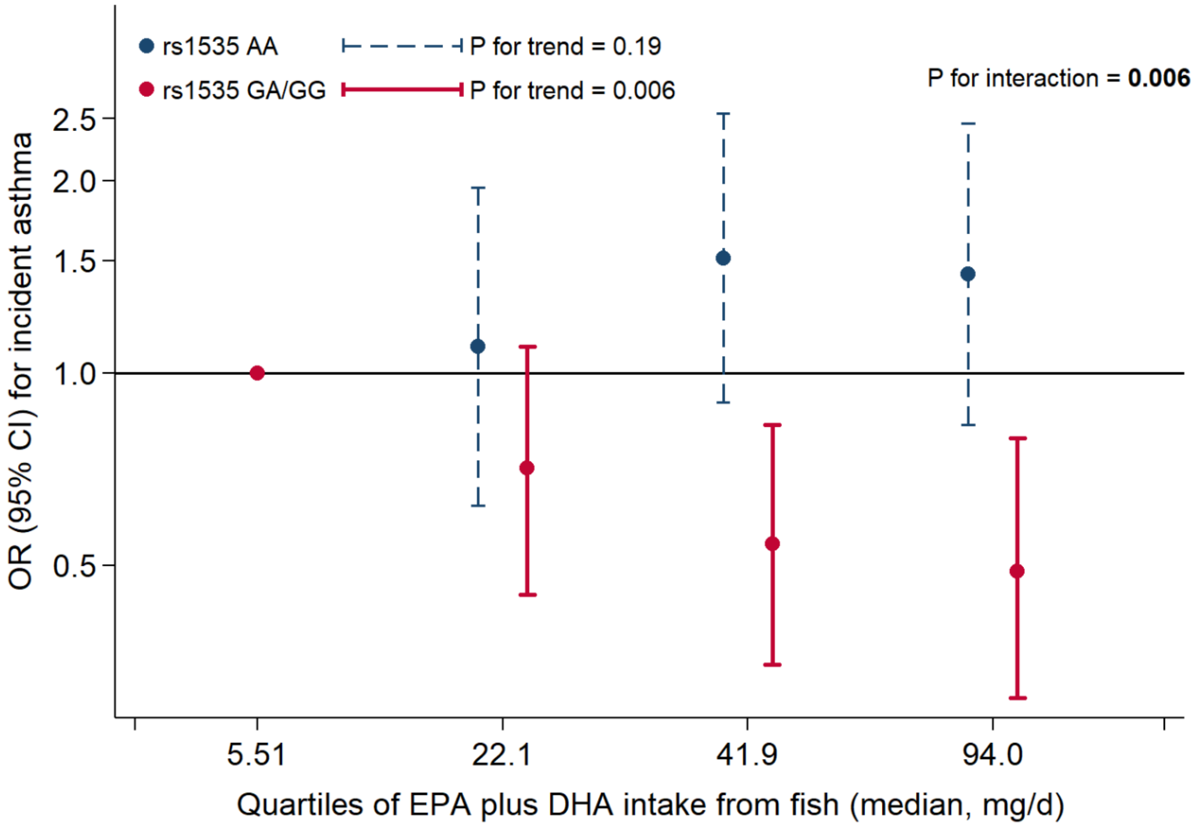

Figure E2: Odds ratio (95% confidence interval) for incident asthma at 11 or 14 years according to intake of EPA plus DHA from fish at 7 years of age, stratified by FADS genotype (rs1535), in ALSPAC

EPA: eicosapentaenoic acid; DHA: docosahexaenoic acid; FADS: fatty acid desaturase.
56.3% of children were in the rs1535 GA/GG genotype group.

Table E1: Participant characteristics according to rs1535 genotype in ALSPAC

	rs1535		
	**AA**	**GA/GG**	**P-value**
n (%)	1607 (44.3)	2024 (55.7)	
EPA plus DHA intake, mg/d	52.15 ± 62.43	49.4 ± 59.35	0.18
Male, n (%)	788 (49.0)	996 (49.2)	0.92
Total energy intake, kJ/day	7578 ± 1701	7578 ± 1672	0.99
BMI, kg/m^2	16.1 ± 1.9	16.1 ± 1.9	0.80
BMI at 13.5 years, kg/m^2	20.1 ± 3.25	20.2 ± 3.28	0.38
Health conscious dietary pattern score	0.05 ± 0.97	-0.02 ± 0.93	0.03
Any supplement use, n (%)	551 (34.3)	670 (33.1)	0.45
Season of dietary information collection, n (%)			0.94
Winter	434 (27.0)	524 (25.9)	
Spring	479 (29.8)	610 (30.1)	
Summer	447 (27.8)	565 (27.9)	
Autumn	233 (14.5)	305 (15.1)	
Missing	14 (0.9)	20 (1.0)	
Breastfeeding at 3 months, n (%)			0.43
Never	208 (12.9)	301 (14.9)	
Stopped/Non-exclusive	750 (46.7)	923 (45.6)	
Exclusive	583 (36.3)	719 (35.5)	
Missing	66 (4.1)	81 (4.0)	
Age at fish introduction, n (%)			0.26
≥9 months	328 (20.4)	421 (20.8)	
6-<9 months	408 (25.4)	559 (27.6)	
<6 months	867 (54.0)	1035 (51.1)	
Missing	4 (0.2)	9 (0.4)	
History of food allergy, n (%)	244 (15.2)	340 (16.8)	0.19
Childcare by day nursery at 15 m			0.40
No, n (%)	1436 (89.4)	1835 (90.7)	
Yes, n (%)	125 (7.8)	141 (7.0)	
Missing	46 (2.9)	48 (2.4)	
Older siblings, n (%)	845 (52.6)	1067 (52.7)	0.94
Younger siblings, n (%)	831 (51.7)	1058 (52.3)	0.74
**Outcomes**			
Incident doctor-diagnosed asthma at 11 or 14 y, n (%)	145 (9.0)	168 (8.3)	0.44

Incident eczema at 11 or 14 y, n (%)	150 (11.1)	180 (10.6)	0.65
Incident hay fever at 11 or 14 y, n (%)	246 (16.8)	307 (16.6)	0.83
**Maternal factors**			
Age, year	29.4 ± 4.3	29.6 ± 4.4	0.17
Education, n (%)			0.29
Secondary or vocational	254 (15.8)	359 (17.7)	
O level	567 (35.3)	705 (34.8)	
A level or degree	774 (48.2)	938 (46.3)	
Missing	12 (0.7)	22 (1.1)	
Housing tenure during pregnancy, n (%)			0.99
Mortgaged/owned	1385 (86.2)	1749 (86.4)	
Council rented	79 (4.9)	98 (4.8)	
Non-council rented	85 (5.3)	102 (5.0)	
Missing	58 (3.6)	75 (3.7)	
Financial difficulty, n (%)			0.99
No	1376 (85.6)	1736 (85.8)	
Yes	225 (14.0)	281 (13.9)	
Missing	6 (0.4)	7 (0.3)	
History of atopy, n (%)			0.65
No	855 (53.2)	1047 (51.7)	
Yes	697 (43.4)	909 (44.9)	
Missing	55 (3.4)	68 (3.4)	
Smoking when child 7 years old, n (%)			0.64
No	1335 (83.1)	1663 (82.2)	
Yes	228 (14.2)	309 (15.3)	
Missing	44 (2.7)	52 (2.6)	
EPA plus DHA intake from fish at 32 w of gestation, mg/d	129 ± 119	128 ± 117	0.88

† Child characteristics pertain to 7 years of age unless otherwise stated.

ALSPAC: Avon Longitudinal Study of Parents and Children; EPA: eicosapentaenoic acid; DHA: docosahexaenoic acid

Table E2: Odds ratio (95% confidence interval) for incident asthma at 11 or 14 years according to intake of arachidonic acid and total n-6 fatty acids at 7 years of age, stratified by child's *FADS* genotype in ALSPAC

	Quartiles of arachidonic acid intake				P for	P for
	**Q1**	**Q2**	**Q3**	**Q4**	**trend***	**interaction**
**Arachidonic acid**						
Median (IQR), mg/d	26.4 (17.1-31.8)	41.4 (38.6-44.4)	54.3 (50.9-58.6)	76.4 (68.6-89.4)		
Cases/non-cases	93/1023	100/1081	101/1070	99/981		
Model 1	1.00	1.00 (0.74-1.35)	1.00 (0.74-1.36)	1.01 (0.73-1.41)	0.94	
Model 2	1.00	1.00 (0.74-1.35)	1.01 (0.74-1.37)	1.01 (0.73-1.42)	0.93	
Model 3	1.00	1.01 (0.75-1.36)	1.01 (0.74-1.38)	1.03 (0.73-1.43)	0.88	
FADS genotype (rs1535): AA						
Cases/non-cases	28/353	43/397	36/374	38/339		
Model 3	1.00	1.36 (0.81-2.27)	1.25 (0.72-2.17)	1.41 (0.78-2.52)	0.34	
FADS genotype (rs1535): GA/GG						
Cases/non-cases	46/451	42/470	40/498	42/438		
Model 3	1.00	0.85 (0.54-1.32)	0.70 (0.44-1.11)	0.75 (0.45-1.25)	0.23	0.19
**Total n-6**						
Median (IQR), g/d	7.35 (6.30-8.14)	9.93 (9.34-10.5)	12.2 (11.6-12.9)	15.6 (14.4-17.2)		
Cases/non-cases	95/1074	112/1077	102/1033	84/971		
Model 1	1.00	1.09 (0.81-1.47)	0.97 (0.70-1.34)	0.76 (0.51-1.13)	0.133	
Model 2	1.00	1.10 (0.81-1.48)	0.97 (0.70-1.35)	0.75 (0.50-1.13)	0.129	
Model 3	1.00	1.10 (0.82-1.49)	0.95 (0.68-1.33)	0.75 (0.50-1.13)	0.118	
FADS genotype (rs1535): AA						
Cases/non-cases	35/372	41/391	37/365	32/335		
Model 3	1.00	1.02 (0.62-1.69)	0.90 (0.52-1.58)	0.77 (0.39-1.52)	.414	
FADS genotype (rs1535): GA/GG						
Cases/non-cases	38/484	50/462	45/476	37/435		
Model 3	1.00	1.25 (0.79-1.99)	0.96 (0.57-1.60)	0.78 (0.42-1.45)	.297	0.93

ALSPAC: Avon Longitudinal Study of Parents and Children; FADS: fatty acid desaturase
* Linear trend was tested by treating the median values of quartiles as a continuous variable
Multivariable model 1: sex and total energy intake at 7 years;
Multivariable model 2: further adjusted for maternal education, housing tenure during pregnancy, financial difficulty during pregnancy, and maternal ethnicity;
Multivariable model 3: further adjusted for maternal history of atopic disease, maternal age at delivery, exclusive breastfeeding, childcare by day nursery at 15 months of age, maternal smoking, older sibling, younger sibling, and season when the FFQ was completed.

Table E3: Odds ratio (95% confidence interval) for incident asthma at 11 or 14 years according to intake of EPA plus DHA from fish at 7 years of age, stratified by child's *FADS* genotype in ALSPAC (sensitivity analyses)

	Quartiles of EPA plus DHA intake from fish				P for	P for
	**Q1**	**Q2**	**Q3**	**Q4**	**trend***	**interaction**
***FADS* genotype (rs1535): AA**						
Median (IQR), mg/d	5.51 (0.00-6.67)	22.1 (22.1-22.1)	39.0 (32.2-48.7)	93.4 (78.6-136)		
Cases/non-cases	26/330	28/322	48/424	43/386		
Model 1	1.00	1.13 (0.63-2.02)	1.55 (0.91-2.64)	1.47 (0.84-2.54)	0.18	
Model 2	1.00	1.11 (0.63-1.97)	1.49 (0.88-2.51)	1.37 (0.79-2.37)	0.28	
Model 3	1.00	1.10 (0.62-1.96)	1.51 (0.90-2.54)	1.45 (0.85-2.49)	0.17	
Model 4	1.00	1.03 (0.57-1.88)	1.25 (0.72-2.17)	1.42 (0.82-2.48)	0.16	
Model 5	1.00	0.87 (0.45-1.67)	1.18 (0.67-2.09)	1.30 (0.73-2.33)	0.23	
Model 6	1.00	1.06 (0.58-1.91)	1.64 (0.96-2.81)	1.58 (0.90-2.78)	0.09	
Model 7	1.00	1.17 (0.63-2.16)	1.75 (1.01-3.05)	1.58 (0.89-2.81)	0.13	
Base model						
Exclusion 1	1.00	1.03 (0.58-1.84)	1.47 (0.87-2.46)	1.38 (0.80-2.37)	0.21	
Exclusion 2	1.00	1.12 (0.59-2.12)	1.47 (0.82-2.62)	1.40 (0.76-2.57)	0.28	
Exclusion 3	1.00	0.95 (0.51-1.76)	1.44 (0.84-2.49)	1.40 (0.80-2.47)	0.17	
Exclusion 4	1.00	1.08 (0.59-1.99)	1.42 (0.82-2.45)	1.33 (0.75-2.36)	0.34	
***FADS* genotype (rs1535): GA/GG**						
Median (IQR), mg/d	5.51 (0.00-6.67)	22.1 (22.1-22.1)	42.5 (32.2-51.5)	94.0 (80.3-141)		
Cases/non-cases	54/419	39/425	43/563	32/449		
Model 1	1.00	0.69 (0.44-1.07)	0.52 (0.33-0.80)	0.47 (0.29-0.76)	0.004	0.005
Model 2	1.00	0.70 (0.45-1.09)	0.53 (0.34-0.82)	0.48 (0.30-0.78)	0.005	0.006
Model 3	1.00	0.71 (0.46-1.11)	0.54 (0.35-0.83)	0.49 (0.31-0.79)	0.006	0.006
Model 4	1.00	0.73 (0.46-1.16)	0.57 (0.36-0.91)	0.48 (0.29-0.79)	0.006	0.005
Model 5	1.00	0.69 (0.42-1.14)	0.56 (0.35-0.91)	0.40 (0.23-0.70)	0.002	0.004
Model 6	1.00	0.76 (0.48-1.20)	0.54 (0.34-0.85)	0.52 (0.31-0.86)	0.014	0.004
Model 7	1.00	0.62 (0.39-0.98)	0.49 (0.31-0.77)	0.46 (0.28-0.75)	0.005	0.003
Base model						
Exclusion 1	1.00	0.71 (0.46-1.10)	0.53 (0.34-0.82)	0.48 (0.30-0.77)	0.004	0.007
Exclusion 2	1.00	0.62 (0.37-1.04)	0.52 (0.31-0.86)	0.51 (0.30-0.89)	0.034	0.02
Exclusion 3	1.00	0.69 (0.43-1.10)	0.59 (0.37-0.93)	0.46 (0.28-0.77)	0.005	0.003
Exclusion 4	1.00	0.68 (0.43-1.08)	0.53 (0.34-0.83)	0.51 (0.31-0.82)	0.01	0.02

IQR: interquartile range; EPA: eicosapentaenoic acid; DHA: docosahexaenoic acid; FADS: fatty acid desaturase

* Linear trend was tested by treating the median values of quartiles as a continuous variable

Base multivariable model: sex and total energy intake at 7 years, maternal education, housing tenure during pregnancy, financial difficulty during pregnancy, and maternal ethnicity, maternal history of atopic disease, maternal age at delivery, exclusive breastfeeding, childcare by day nursery at 15 months of age, maternal smoking, older sibling, younger sibling, and season when the FFQ was completed;

Multivariable model 1: further adjusted for age at first exposure to fish;

Multivariable model 2: further adjusted for health-conscious dietary pattern;

Multivariable model 3: further adjusted for any supplement use;

Multivariable model 4: further adjusted for BMI at 7 years;

Multivariable model 5: further adjusted for BMI at 14 years;

Multivariable model 6: further adjusted for maternal intake of EPA plus DHA from fish during pregnancy;

Multivariable model 7: further adjusted for the first 10 principal components (PC) derived by PC analysis from ALSPAC genome-wide data.

Exclusions: 1) children of non-white mothers, 2) children with a history of food allergy, 3) children with extreme energy intakes or users of fish liver oil or omega-3 supplements, 4) children with wheeze at 7 years

Table E4: Odds ratio (95% confidence interval) for incident asthma at 11 or 14 years according to intake of EPA from fish at 7 years of age, stratified by child's *FADS* genotype in ALSPAC

	Quartiles of EPA intake from fish				P for	P for
	**Q1**	**Q2**	**Q3**	**Q4**	**trend***	**interaction**
Median (IQR), mg/d	2.55 (0.00-3.53)	10.2 (6.97-10.2)	14.1 (12.9-16.4)	34.4 (27.9-47.0)		
Cases/non-cases	105/1005	91/1056	99/1038	95/1054		
Model 1	1.00	0.81 (0.61-1.09)	0.89 (0.67-1.19)	0.84 (0.62-1.12)	0.39	
Model 2	1.00	0.81 (0.61-1.10)	0.89 (0.66-1.19)	0.84 (0.62-1.13)	0.39	
Model 3	1.00	0.84 (0.62-1.13)	0.89 (0.67-1.20)	0.84 (0.63-1.14)	0.39	
FADS genotype (rs1535): AA						
Cases/non-cases	28/348	34/362	42/362	41/391		
Model 3	1.00	1.19 (0.70-2.04)	1.49 (0.88-2.51)	1.29 (0.76-2.19)	0.50	
FADS genotype (rs1535): GA/GG						
Cases/non-cases	58/443	39/478	38/483	33/453		
Model 3	1.00	0.59 (0.38-0.91)	0.56 (0.36-0.87)	0.50 (0.32-0.80)	0.01	0.03

ALSPAC: Avon Longitudinal Study of Parents and Children; EPA: eicosapentaenoic acid; FADS: fatty acid desaturase

* Linear trend was tested by treating the median values of quartiles as a continuous variable

Multivariable model 1: sex and total energy intake at 7 years;

Multivariable model 2: further adjusted for maternal education, housing tenure during pregnancy, financial difficulty during pregnancy, and maternal ethnicity;

Multivariable model 3: further adjusted for maternal history of atopic disease, maternal age at delivery, exclusive breastfeeding, childcare by day nursery at 15 months of age, maternal smoking, older sibling, younger sibling, and season when the FFQ was completed.

Table E5: Odds ratio (95% confidence interval) for incident asthma at 11 or 14 years according to intake of DHA from fish at 7 years of age, stratified by child's *FADS* genotype in ALSPAC

	**Quartiles of DHA intake from fish**				**P for**	**P for**
	**Q1**	**Q2**	**Q3**	**Q4**	**trend***	**interaction**
Median (IQR), mg/d	2.96 (0.00-5.69)	11.9 (11.9-11.9)	25.7 (18.7-34.6)	63.8 (48.8-85.4)		
Cases/non-cases	98/938	79/925	117/1211	96/1079		
Model 1	1.00	0.80 (0.59-1.10)	0.91 (0.68-1.20)	0.82 (0.61-1.11)	0.40	
Model 2	1.00	0.81 (0.59-1.10)	0.90 (0.68-1.20)	0.82 (0.61-1.11)	0.38	
Model 3	1.00	0.82 (0.60-1.13)	0.91 (0.68-1.21)	0.83 (0.61-1.13)	0.40	
FADS genotype (rs1535): AA						
Cases/non-cases	25/326	29/315	48/429	43/393		
Model 3	1.00	1.22 (0.69-2.17)	1.55 (0.91-2.62)	1.46 (0.85-2.52)	0.25	
FADS genotype (rs1535): GA/GG						
Cases/non-cases	53/412	36/416	48/560	31/469		
Model 3	1.00	0.67 (0.43-1.06)	0.61 (0.40-0.93)	0.46 (0.29-0.75)	0.004	0.008

ALSPAC: Avon Longitudinal Study of Parents and Children; DHA: docosahexaenoic acid, FADS: fatty acid desaturase

* Linear trend was tested by treating the median values of quartiles as a continuous variable

Multivariable model 1: sex and total energy intake at 7 years;

Multivariable model 2: further adjusted for maternal education, housing tenure during pregnancy, financial difficulty during pregnancy, and maternal ethnicity;

Multivariable model 3: further adjusted for maternal history of atopic disease, maternal age at delivery, exclusive breastfeeding, childcare by day nursery at 15 months of age, maternal smoking, older sibling, younger sibling, and season when the FFQ was completed.

Table E6: Odds ratio (95% confidence interval) for incident asthma at 11 or 14 years according to intake of EPA plus DHA from fish at 7 years of age adjusted for total energy intake and applying inverse probability weighting, stratified by child's *FADS* genotype in ALSPAC

	Quartiles of EPA plus DHA intake from fish				P for	P for
	**Q1**	**Q2**	**Q3**	**Q4**	**trend***	**interaction**
**Energy adjusted intakes using residual method**						
FADS genotype (rs1535): AA						
Median (IQR), mg/d	7.88 (2.68-12.1)	23.5 (20.0-27.6)	43.2 (36.6-53.3)	93.5 (77.7-134)		
Cases/non-cases	33/354	32/348	39/374	41/386		
Model 3	1.00	1.08 (0.64-1.83)	1.26 (0.76-2.10)	1.23 (0.74-2.03)	0.42	
FADS genotype (rs1535): GA/GG						
Median (IQR), mg/d	7.81 (1.89-12.4)	23.8 (19.7-27.8)	45.3 (37.6-54.6)	95.1 (79.0-137)		
Cases/non-cases	55/437	41/491	42/467	30/461		
Model 3	1.00	0.66 (0.42-1.03)	0.72 (0.46-1.12)	0.50 (0.31-0.80)	0.01	0.02
**Inverse probability weighing**						
FADS genotype (rs1535): AA						
Model 3	1.00	1.18 (0.60-2.35)	1.51 (0.81-2.79)	1.60 (0.83-3.09)	0.15	
FADS genotype (rs1535): GA/GG						
Model 3	1.00	0.58 (0.34-0.99)	0.61 (0.36-1.03)	0.55 (0.31-0.98)	0.05	0.03

ALSPAC: Avon Longitudinal Study of Parents and Children; EPA: eicosapentaenoic acid; DHA: docosahexaenoic acid; FADS: fatty acid desaturase

* Linear trend was tested by treating the median values of quartiles as a continuous variable

Multivariable model 1: sex and total energy intake at 7 years;

Multivariable model 2: further adjusted for maternal education, housing tenure during pregnancy, financial difficulty during pregnancy, and maternal ethnicity;

Multivariable model 3: further adjusted for maternal history of atopic disease, maternal age at delivery, exclusive breastfeeding, childcare by day nursery at 15 months of age, maternal smoking, older sibling, younger sibling, and season when the FFQ was completed.

Table E7: Odds ratio (95% confidence interval) for incident asthma at 11 or 14 years according to maternal intake of EPA plus DHA from fish at 32 weeks of gestation, stratified by maternal *FADS* genotype in ALSPAC

	Quartiles of EPA plus DHA intake from fish				P for	P for
	Q1	Q2	Q3	Q4	trend*	interaction
Median (IQR), g/d	0.0 (0.0-0.0)	37.9 (18.8-69.1)	88.2 (75.1-126)	277 (239-296)		
Cases/non-cases	55/431	126/1354	112/1237	108/1253		
Model 1	1.00	0.74 (0.53-1.04)	0.73 (0.52-1.03)	0.70 (0.49-0.99)	0.26	
Model 2	1.00	0.72 (0.51-1.01)	0.73 (0.51-1.04)	0.69 (0.48-0.98)	0.27	
Maternal *FADS* genotype (rs1535): AA						
Cases/non-cases	18/147	43/449	36/414	36/398		
Model 2	1.00	0.73 (0.40-1.35)	0.76 (0.41-1.43)	0.74 (0.39-1.42)	0.73	
Maternal *FADS* genotype (rs1535): GA/GG						
Cases/non-cases	19/178	57/548	47/500	36/540		
Model 2	1.00	0.96 (0.55-1.69)	0.95 (0.53-1.69)	0.68 (0.37-1.26)	0.10	0.34

IQR: interquartile range; EPA: eicosapentaenoic acid; DHA: docosahexaenoic acid; FADS: fatty acid desaturase

* Linear trend was tested by treating the median values of quartiles as a continuous variable

Multivariable model 1: age and total energy intake;

Multivariable model 2: further adjusted for maternal education, housing tenure during pregnancy, financial difficulty during pregnancy, ethnicity, history of atopic disease, age at delivery, smoking during pregnancy, parity, multiple pregnancy, sex of child, season of birth, breastfeeding duration, infections, antibiotics and paracetamol use during pregnancy, and anxiety.

Table E8: Odds ratio (95% confidence interval) for incident asthma at 11 or 14 years according to intake of EPA plus DHA from fish at 7 years of age, stratified by other child polymorphisms related to n-3 fatty acid metabolism (those not in linkage disequilibrium with each other†) in ALSPAC

	Quartiles of EPA plus DHA intake from fish				**P for**	**P for**
**SNP† (gene)**	**Q1**	**Q2**	**Q3**	**Q4**	**trend***	**interaction**
**rs780094** (GCKR)						
TT: Cases/non-cases	8/120	12/112	16/139	13/111		
Model 3	1.00	1.83 (0.67-4.97)	1.35 (0.52-3.53)	1.66 (0.63-4.42)	0.49	
TC/CC: Cases/non-cases	72/629	55/635	75/848	62/724		
Model 3	1.00	0.74 (0.51-1.08)	0.75 (0.53-1.06)	0.71 (0.49-1.02)	0.14	0.13
**rs3734398** (ELOVL2)						
TT: Cases/non-cases	11/228	31/307	26/338	22/271		
Model 3	1.00	2.18 (1.05-4.54)	1.45 (0.68-3.10)	1.47 (0.67-3.23)	0.97	
CT/CC: Cases/non-cases	59/443	60/623	55/543	49/567		
Model 3	1.00	0.74 (0.50-1.10)	0.77 (0.52-1.15)	0.62 (0.41-0.94)	0.05	0.15
**rs12662634** (ELOVL2-AS1)						
GG: Cases/non-cases	37/451	61/643	57/635	43/564		
Model 3	1.00	1.15 (0.75-1.78)	1.02 (0.66-1.60)	0.85 (0.53-1.37)	0.29	
AG/AA: Cases/non-cases	33/220	30/287	24/246	28/274		
Model 3	1.00	0.68 (0.40-1.18)	0.61 (0.35-1.09)	0.65 (0.37-1.14)	0.21	0.54
**rs174448** (between FADS2 and FADS3)						
AA: Cases/non-cases	27/263	37/371	43/379	34/354		
Model 3	1.00	1.06 (0.62-1.82)	1.22 (0.72-2.09)	0.97 (0.55-1.70)	0.87	
GA/GG: Cases/non-cases	37/384	51/528	36/475	35/458		
Model 3	1.00	0.98 (0.62-1.55)	0.73 (0.45-1.20)	0.72 (0.44-1.18)	0.12	0.41
**rs11693320** (DPP10)						
AA: Cases/non-cases	47/415	44/578	46/568	44/531		
Model 3	1.00	0.70 (0.45-1.08)	0.69 (0.44-1.07)	0.72 (0.46-1.12)		
GA/GG: Cases/non-cases	20/226	44/323	32/273	26/277		
Model 3	1.00	1.43 (0.80-2.53)	1.23 (0.67-2.26)	0.89 (0.47-1.70)	0.30	0.98
**rs174602** (FADS2)						
TT: Cases/non-cases	29/332	44/461	53/458	37/438		
Model 3	1.00	1.07 (0.64-1.77)	1.21 (0.74-1.99)	0.87 (0.52-1.48)	0.51	
CT/CC: Cases/non-cases	16/161	23/211	16/212	16/190		
Model 3	1.00	1.18 (0.58-2.39)	0.82 (0.38-1.76)	0.81 (0.37-1.77)	0.39	0.49
**rs174450** (FADS3)						
TT: Cases/non-cases	13/195	30/254	29/251	21/245		
Model 3	1.00	1.96 (0.97-3.96)	1.97 (0.96-4.04)	1.34 (0.63-2.86)	0.99	
GT/GG: Cases/non-cases	47/446	56/628	47/581	47/548		
Model 3	1.00	0.82 (0.55-1.25)	0.72 (0.46-1.11)	0.76 (0.49-1.18)	0.26	0.51

ALSPAC: Avon Longitudinal Study of Parents and Children; EPA: eicosapentaenoic acid; DHA: docosahexaenoic acid; FADS: fatty acid desaturase

Except for rs780094, carriers of the minor allele (heterozygotes and homozygotes for the minor allele) were combined either because minor alleles were associated with a lower concentration of long-chain

PUFA (5 SNPs), or because a low minor allele frequency resulted in insufficient statistical power (rs11693320).

[†] The SNPs in linkage disequilibrium with rs1535 were not presented: rs174547, rs174538, rs174535, rs174575, rs174556, rs174537, rs174576, rs174545, rs174561, rs174583. The SNPs in linkage disequilibrium with rs3734398 were also not presented: rs3798713 and rs2236212.

[*] Linear trend was tested by treating the median values of quartiles as a continuous variable

Multivariable model 1: sex and total energy intake at 7 years;

Multivariable model 2: further adjusted for maternal education, housing tenure during pregnancy, financial difficulty during pregnancy, and maternal ethnicity;

Multivariable model 3: further adjusted for maternal history of atopic disease, maternal age at delivery, exclusive breastfeeding, childcare by day nursery at 15 months of age, maternal smoking, older sibling, younger sibling, and season when the FFQ was completed.

Table E9. Participant* characteristics in the BAMSE cohort

	N	%
**Male sex**	1080	50.5
**Maternal education**		
9-year compulsory school	134	6.3
2-year secondary school	512	24.0
3-4-year secondary school	554	25.9
University or college	928	43.4
**Parental occupation**		
Blue collar	293	13.7
Lower white collar	959	44.9
Higher white collar	865	40.5
Other	17	0.8
**Maternal ethnicity: European**	2032	95.0
**Maternal history of atopic disease**	523	24.5
**Exclusive breastfeeding 4 months or more**	1739	81.3
**Childcare by day nursery at 2 years of age**	1574	73.7
**Maternal smoking when the child was 8 years of age**		
None	1847	88.0
1-9 cigarettes/day	112	5.3
10-19 cigarettes/day	114	5.4
≥20/day	26	1.2
**Older siblings**	994	46.5
**Season of dietary information collection**		
Winter	451	21.1
Spring	868	40.6
Summer	178	8.3
Autumn	641	30.0
	**Mean**	**SD**
**Maternal age at delivery, years**	31.0	4.4
**Total energy intake, kcal/day**	1913	463

* Children included in incident asthma analysis (n=2,138).
BAMSE: Swedish abbreviation for Children, Allergy, Milieu, Stockholm, Epidemiology
Numbers may not add up to the total, due to missing data.

Table E10. Intakes of fish, and EPA and DHA from fish, in BAMSE and ALSPAC.

	**BAMSE** (n=2,138*)			**ALSPAC** (n=4,543*)		
	**Median**	**IQR**	**Min-Max**	**Median**	**IQR**	**Min-Max**
**Fish intake, times/wk**						
Total fish	1.9	1.2	0-15	2.5	1-4	0-42
Oily fish	0.5	0.5	0-7	0	0-0	0-10
**n-3 PUFA intake from fish, mg/d**						
EPA	41.0	45.4	0-822	11.2	5.99-24.0	0-307
DHA	104	116	0-2266	17.6	9.77-41.4	0-519

BAMSE: Swedish abbreviation for Children, Allergy, Milieu, Stockholm, Epidemiology; ALSPAC: Avon Longitudinal Study of Parents and Children; IQR: interquartile range; PUFA: polyunsaturated fatty acid; EPA: eicosapentaenoic acid; DHA: docosahexaenoic acid

* Children with data on nutrient and fish intake at age 7 (ALSPAC) and 8 (BAMSE) years and data on incident asthma till 14 (ALSPAC) and 16 (BAMSE) years.

Table E11: Odds ratio (95% confidence interval) for incident asthma at 12 or 16 years of age, according to intake of EPA and DHA from fish at 8 years of age, stratified by *FADS* genotype in BAMSE

	Quartiles of EPA and DHA intake from fish				P for	P for
	**Q1**	**Q2**	**Q3**	**Q4**	**trend***	**interaction**
**EPA**						
Median (IQR), mg/d	9.9 (8.4)	27.0 (13.4)	50.3 (10.6)	84.7 (33.3)		
Cases/non-cases	43/479	40/481	34/520	33/507		
Model 1	1.00	0.93 (0.59-1.45)	0.72 (0.45-1.15)	0.71 (0.44-1.14)	0.11	
Model 2	1.00	0.93 (0.59-1.46)	0.72 (0.45-1.15)	0.66 (0.40-1.07)	0.06	
Model 3	1.00	0.99 (0.63-1.56)	0.71 (0.43-1.14)	0.63 (0.38-1.05)	0.04	
FADS genotype (rs1535): AA						
Cases/non-cases	14/165	15/173	14/159	15/171		
Model 3	1.00	1.08 (0.49-2.39)	0.94 (0.41-2.16)	0.92 (0.40-2.11)	0.77	
FADS genotype (rs1535): GA/GG						
Cases/non-cases	24/261	22/262	20/311	15/272		
Model 3	1.00	1.01 (0.54-1.90)	0.71 (0.37-1.35)	0.56 (0.27-1.15)	0.07	0.05
**DHA**						
Median (IQR), mg/d	24.1 (20.4)	62.9 (33.1)	127.4 (28.2)	207.5 (82.7)		
Cases/non-cases	45/477	38/485	35/512	32/513		
Model 1	1.00	0.83 (0.53-1.30)	0.72 (0.45-1.14)	0.64 (0.40-1.04)	0.07	
Model 2	1.00	0.83 (0.53-1.30)	0.71 (0.45-1.13)	0.59 (0.36-0.97)	0.03	
Model 3	1.00	0.87 (0.55-1.37)	0.70 (0.43-1.13)	0.55 (0.33-0.92)	0.02	
FADS genotype (rs1535): AA						
Cases/non-cases	16/162	12/177	15/168	15/161		
Model 3	1.00	0.70 (0.31-1.56)	0.83 (0.38-1.84)	0.81 (0.36-1.83)	0.80	
FADS genotype (rs1535): GA/GG						
Cases/non-cases	24/261	23/262	20/296	14/287		
Model 3	1.00	1.03 (0.55-1.91)	0.74 (0.39-1.41)	0.48 (0.23-0.99)	0.03	0.02

BAMSE: Swedish abbreviation for Children, Allergy, Milieu, Stockholm, Epidemiology; IQR: interquartile range; EPA: eicosapentaenoic acid; DHA: docosahexaenoic acid; FADS: fatty acid desaturase

* Linear trend was tested by treating the median values of quartiles as a continuous variable

Multivariable model 1: sex and total energy intake at 8 years;

Multivariable model 2: further adjusted for maternal education, parental occupation and maternal ethnicity;

Multivariable model 3: further adjusted for maternal history of atopic disease, maternal age at delivery, exclusive breastfeeding, childcare by day nursery at 2 years of age, maternal smoking, older sibling, and season when the FFQ was completed.
