# Supplementary material for: Intake of n-3 polyunsaturated fatty acids in childhood, FADS genotype and incident asthma
Source: Eur Respir J. 2021 Sep 2;58(3):2003633. doi: 10.1183/13993003.03633-2020 (PMC8411098; doi:10.1183/13993003.03633-2020)
Supplement: Supplementary file 2 [file ERJ-03633-2020.Shareable.pdf]

EUROPEAN RESPIRATORY JOURNAL
ORIGINAL RESEARCH ARTICLE

Intake of *n*-3 polyunsaturated fatty acids in childhood, *FADS* genotype and incident asthma

SHAREABLE PDF

Mohammad Talaei [1], Emmanouela Sdona [2], Philip C. Calder [3,4], Louise R. Jones [5], Pauline M. Emmett [5], Raquel Granell [6], Anna Bergström [2,7], Erik Melén [2,8,9] and Seif O. Shaheen [1]

[1]Institute of Population Health Sciences, Barts and The London School of Medicine and Dentistry, Queen Mary University of London, London, UK. [2]Institute of Environmental Medicine, Karolinska Institute, Stockholm, Sweden. [3]Human Development and Health, Faculty of Medicine, University of Southampton, Southampton, UK. [4]NIHR Southampton Biomedical Research Centre, University Hospital Southampton NHS Foundation Trust and University of Southampton, Southampton, UK. [5]Centre for Academic Child Health, Population Health Sciences, Bristol Medical School, University of Bristol, Bristol, UK. [6]MRC Integrative Epidemiology Unit, Population Health Sciences, Bristol Medical School, University of Bristol, Bristol, UK. [7]Centre for Occupational and Environmental Medicine, Region Stockholm, Stockholm, Sweden. [8]Dept of Clinical Science and Education, Södersjukhuset, Karolinska Institute, Stockholm, Sweden. [9]Sachs' Children and Youth Hospital, Södersjukhuset, Stockholm, Sweden.

Corresponding author: Mohammad Talaei (m.talaei@qmul.ac.uk)

Check for updates

Shareable abstract (@ERSpublications)
In children with a common fatty acid desaturase (*FADS*) variant, higher intake of eicosapentaenoic acid (EPA) and docosahexaenoic acid (DHA) from fish in mid-childhood was strongly associated with a lower risk of incident asthma up to mid-adolescence https://bit.ly/2M4VxO6

Cite this article as: Talaei M, Sdona E, Calder PC, *et al*. Intake of *n*-3 polyunsaturated fatty acids in childhood, *FADS* genotype and incident asthma. *Eur Respir J* 2021; 58: 2003633 [DOI: 10.1183/13993003.03633-2020].

This single-page version can be shared freely online.

Copyright ©The authors 2021

This version is distributed under the terms of the Creative Commons Attribution Licence 4.0.

This article has supplementary material available from erj.ersjournals.com

This article has an editorial commentary: https://doi.org/10.1183/13993003.01386-2021

Received: 25 Sept 2020
Accepted: 14 Jan 2021

Abstract

Longitudinal evidence on the relation between dietary intake of *n*-3 (ω-3) very-long-chain polyunsaturated fatty acids, *i.e.* eicosapentaenoic acid (EPA) and docosahexaenoic acid (DHA), in mid-childhood and asthma risk is scarce. We aimed to investigate whether a higher intake of EPA and DHA from fish in childhood is associated with a lower risk of incident asthma.

In the Avon Longitudinal Study of Parents and Children, dietary intakes of EPA and DHA from fish were estimated by food frequency questionnaire at 7 years of age. We used logistic regression, controlling for confounders, to analyse associations between intake of EPA and DHA (quartiles) and incidence of doctor-diagnosed asthma at age 11 or 14 years, and explored potential effect modification by a fatty acid desaturase (*FADS*) polymorphism (rs1535). Replication was sought in the Swedish BAMSE birth cohort.

There was no evidence of association between intake of EPA plus DHA from fish and incident asthma overall (n=4543). However, when stratified by *FADS* genotype, the odds ratio comparing the top *versus* bottom quartile among the 2025 minor G allele carriers was 0.49 (95% CI 0.31–0.79; p_{trend}=0.006), but no inverse association was observed in the homozygous major A allele group (OR 1.43, 95% CI 0.83–2.46; p_{trend}=0.19) ($p_{interaction}$=0.006). This gene–nutrient interaction on incident asthma was replicated in BAMSE.

In children with a common *FADS* variant, higher intake of EPA and DHA from fish in childhood was strongly associated with a lower risk of incident asthma up to mid-adolescence.

cc BY
